# Marine Collagen Hydrolysates Downregulate the Synthesis of Pro-Catabolic and Pro-Inflammatory Markers of Osteoarthritis and Favor Collagen Production and Metabolic Activity in Equine Articular Chondrocyte Organoids

**DOI:** 10.3390/ijms22020580

**Published:** 2021-01-08

**Authors:** Bastien Bourdon, Romain Contentin, Frédéric Cassé, Chloé Maspimby, Sarah Oddoux, Antoine Noël, Florence Legendre, Nicolas Gruchy, Philippe Galéra

**Affiliations:** 1 Normandie University, UNICAEN, BIOTARGEN, 14000 Caen, France; bastien.bourdon@unicaen.fr (B.B.); contentinr@email.chop.edu (R.C.); frederic.casse@unicaen.fr (F.C.); chloe.maspimby@outlook.com (C.M.); florence.legendre@unicaen.fr (F.L.); nicolas.gruchy@unicaen.fr (N.G.); 2 Dielen Laboratory, 50110 Tourlaville, France; sarah-oddoux@dielen.fr (S.O.); antoine-noel@dielen.fr (A.N.); 3 Department of Genetics, Normandy Center for Genomic and Personalized Medicine, Caen University Hospital, 14000 Caen, France

**Keywords:** chondrocyte, equine model, matrix-associated autologous chondrocyte implantation (MACI), osteoarthritis, collagen, collagen hydrolysates, interleukin-1, catabolic markers, senescence, in vitro repair

## Abstract

Articular cartilage experiences mechanical constraints leading to chondral defects that inevitably evolve into osteoarthritis (OA), because cartilage has poor intrinsic repair capacity. Although OA is an incurable degenerative disease, several dietary supplements may help improve OA outcomes. In this study, we investigated the effects of Dielen^®^ hydrolyzed fish collagens from skin (Promerim^®^30 and Promerim^®^60) and cartilage (Promerim^®^40) to analyze the phenotype and metabolism of equine articular chondrocytes (eACs) cultured as organoids. Here, our findings demonstrated the absence of cytotoxicity and the beneficial effect of Promerim^®^ hydrolysates on eAC metabolic activity under physioxia; further, Promerim^®^30 also delayed eAC senescence. To assess the effect of Promerim^®^ in a cartilage-like tissue, eACs were cultured as organoids under hypoxia with or without BMP-2 and/or IL-1β. In some instances, alone or in the presence of IL-1β, Promerim^®^30 and Promerim^®^40 increased protein synthesis of collagen types I and II, while decreasing transcript levels of proteases involved in OA pathogenesis, namely *Htra1*, and the metalloproteinases *Mmp1-3*, *Adamts5*, and *Cox2*. Both Promerim^®^ hydrolysates also decreased Htra1 protein amounts, particularly in inflammatory conditions. The effect of Promerim^®^ was enhanced under inflammatory conditions, possibly due to a decrease in the synthesis of inflammation-associated molecules. Finally, Promerim^®^ favored in vitro repair in a scratch wound assay through an increase in cell proliferation or migration. Altogether, these data show that Promerim^®^30 and 40 hold promise as dietary supplements to relieve OA symptoms in patients and to delay OA progression.

## 1. Introduction

Articular cartilage is a highly specialized tissue covering the extremities of bones, thereby ensuring smooth joint movements as well as shock absorption. Hyaline articular cartilage is composed of an abundant extracellular matrix (ECM) and a main cell type, the chondrocyte, which is present in small quantities (1 to 2% of the cartilage volume) [1]. Chondrocytes synthesize an ECM composed of characteristic molecules, such as type II, IX, XI collagens and aggrecan, especially during embryonic stages, although these molecules also ensure the homeostasis of the tissue in adulthood, albeit with low turnover [2]. Hyaline cartilage synthesis can be promoted by growth factors, such as transforming growth factor-ß (TGF-ß) or bone morphogenic proteins (BMP) [3]. Cartilage is not innervated and is avascular; for this reason, chondrocytes subsist in hypoxia [4]. Oxygen is delivered to the cartilage via the synovial fluid, in part secreted by synoviocytes, or through diffusion from the subchondral bones; oxygen concentrations range from 1 to 10% depending on the depth of the tissue [5].

One of the hallmarks of osteoarthritis (OA) is the disruption of cartilage homeostasis [6,7,8,9], further aggravated by the poor intrinsic capacity of cartilage for self-repair [10]. Subsequently, cartilage degradation occurs, eventually exposing the subchondral bone, and leading to stiff and painful joints. In OA, catabolism is increased and anabolism is downregulated. Nevertheless, the first response following cartilage injury is a transient increase in chondrocyte proliferation, whereby chondrocytes form clusters and enhance their ECM production, particularly by synthesizing type I collagen [6,7,9]. However, type I collagen does not share the same biomechanical properties as typical cartilage collagens, and thus makes for a brittle cartilage matrix called fibrocartilage. Fibrocartilage no longer fulfills hyaline cartilage function and degrades more quickly. In an OA pro-inflammatory context, cytokines such as interleukin-1 (IL-1) and the tumor necrosis factor-alpha (TNF-alpha) cause tissue degradation by decreasing anabolism and inducing the expression/activation of proteases including metalloproteinases (MMPs) and aggrecanases [6,7]. Although the OA etiology is diverse, it is generally due to repeated shocks, and/or joint overload, combined with ageing. Other factors such as obesity and genetic factors may also be related to OA.

Humans and horses have very similar cartilage in many ways, particularly in terms of their biochemical composition, cell composition, and the thickness of articular cartilage [11]. In addition, like humans, horses can develop OA spontaneously. Moreover, humans and sport horses can experience comparable biomechanical stresses, and the causes of the onset of the OA process are very similar, namely sports activities and ageing. Numerous studies aimed at improving OA treatments in the equine model are therefore directly transposable to humans, and the horse represents the best large mammal model for pre-clinical studies in the context of osteo-articular disorders, particularly arthropathies. Additionally, the size of horse joints makes the horse an ideal model from an experimental point of view, because a large amount of cartilage can be recovered and in vivo experiments can be carried out in the horse. Joint injuries also represent the main cause of the decrease in physical performance and lameness in horses [12]. They generally lead to the premature cessation of the horse’s sport career, incurring significant financial losses for the equine industry [13].

To date, there are no long-term solutions to treat OA, either in humans or in horses. For the management of OA, the use of a combination of pharmacological and non-pharmacological treatments is highly recommended. The main pharmacological treatments aim to reduce inflammation and pain through the use of non-steroidal anti-inflammatory drugs (NSAID) and analgesics. For severe OA, intra-articular injections of corticosteroids or hyaluronan can be prescribed and, in the final stages of OA, especially in humans, only prosthetic surgery is efficient [14,15,16]. The use of nutraceuticals, or dietary supplements, represents an ongoing strategy for the management and prevention of OA as a complement to classical clinical therapies [17].

Horse owners are becoming increasingly interested in alternatives to traditional clinical approaches, such as nutritional supplements [18]. Numerous dietary supplements, such as glucosamine, chondroitin sulfate, and collagen hydrolysates represent promising therapeutic alternatives to favoring cartilage homeostasis when they are used alone or in combination [17,19,20,21,22,23]. The most common products used as nutraceuticals are chondroitin sulfate and glucosamine [17]. One study demonstrated that chondroitin sulfate improves pain symptoms in the short term, with a low risk of adverse events [22]. Chondrocyte culture in the presence of glucosamine may promote the production of proteoglycans [19]. Other food supplements, such as curcumin or frankincense (*Boswellia* sp.), also appear to hold promise [17,20,21]. Dietary supplements can be combined with surgical techniques to help patients to recover properly. However, to date, no food supplement has been demonstrated to be able to regenerate hyaline articular cartilage.

Matrikines, which are bioactive peptides resulting from the degradation of the ECM, may be promising therapeutic possibilities for OA, because some of them have been described as playing a role in wound healing and in the synthesis of anabolic components in fibroblasts [24]. Other studies have shown that, in women with moderate knee osteoarthritis, daily oral intake of collagen peptides for 6 months reduces pain and increases mobility of the affected joint [25]. Promerim^®^30, made of fish skin collagens, is a main ingredient in the dietary supplement Osteocalm^®^, which has proven, like other collagens (such as undenatured type II collagen (UC-II^®^)), to be clinically effective for treating joint pain and stiffness associated with OA in humans [17,26,27]. Moreover, recent investigations show that enzymatically hydrolyzed collagens are absorbed and distributed to joint tissues and have analgesic and anti-inflammatory properties [28].

In the present study, we assessed the effects of three collagen hydrolysates of marine origin (derived from fish skin (Promerim^®^30 and Promerim^®^60) and fish cartilage (Promerim^®^40)) on the metabolism of chondrocytes cultured as cartilage organoids, as previously described [9,29,30]. The cartilaginous quality of the organoids and the effects of Promerim^®^ were assessed through an extensive study of transcripts and protein amounts of typical and atypical markers of hyaline chondrocytes. Additionally, we characterized the effect of Promerim^®^ on chondrocyte proliferation/viability and senescence. This study attempted to identify some of the molecular mechanisms that may be responsible for the potential beneficial effects of hydrolyzed collagens on chondrocyte metabolism.

## 2. Results

### 2.1. Promerim^®^30, 40, and 60 Have no Cytotoxic Effects on Equine Articular Chondrocytes, They Promote Their Metabolic Activity and Promerim^®^30 Downregulates Cellular Senescence

**Figure 1:**
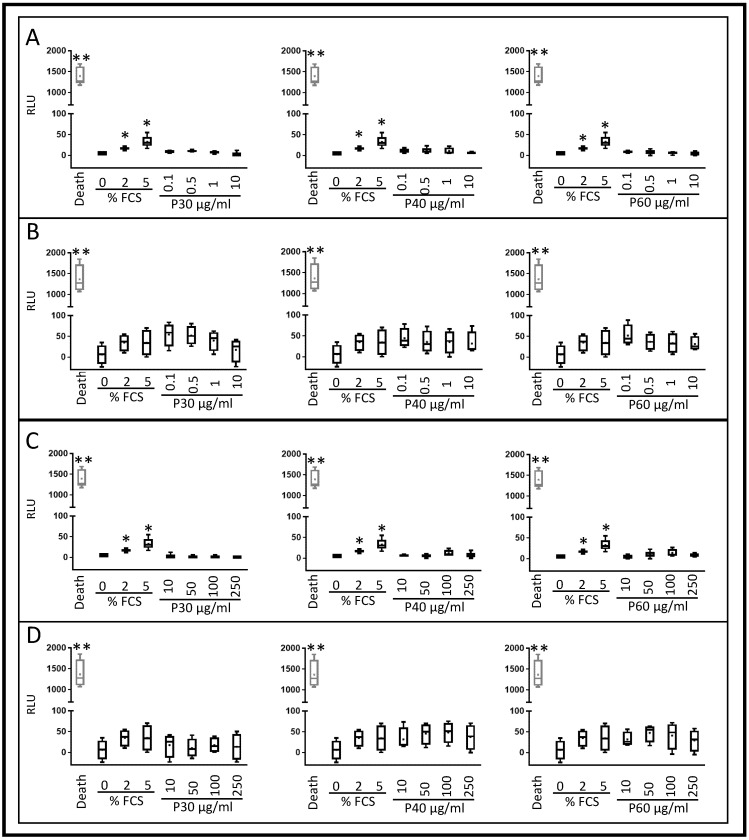
Promerim^®^30, 40, and 60 have no cytotoxic effect on equine articular chondrocytes cultured in hypoxia. Equine articular chondrocytes were amplified and seeded at P3. At 80% of confluency, the cells were treated with Promerim^®^ hydrolysates at several concentrations (0.1, 0.5, 1, and 10 μg/mL) (**A**,**B**) and (10, 50, 100, 250 μg/mL) (**C**,**D**) in the absence (0% (**A**,**C**)) or presence of 2% fetal calf serum (FCS) (**B**,**D**) and then cultured for 72 h in hypoxia. Controls with 0, 2, and 5% of FCS were included, as well as a death control (triton-induced death). The levels of adenylate kinase were measured in the media after 72 h of culture (Toxilight kit, Interchim). Data are represented as box plots (*n* = 5). Statistical analyses were performed using the Mann–Whitney test (* *p* < 0.05, ** *p* < 0.01),and the 2% and 0% FCS conditions were used as references. P30, P40, and P60: Promerim^®^30, Promerim^®^40, and Promerim^®^60.

eACs were cultured as cell monolayers. At 80% confluency, Promerim^®^30, 40, or 60 was added at concentrations ranging from 0.1 µg/mL to 250 µg/mL. Then, eACs were grown either in normoxia or in hypoxia, in serum-free medium, or in the presence of 2% FCS for 72 h. Hypoxia and the serum-free media were used to mimic the in vivo microenvironment of chondrocytes as much as possible. Regardless of the oxic condition or the FCS concentration, none of the Promerim^®^ hydrolysates, regardless of concentration, were cytotoxic for eACs (Figure 1 and Figure S1).

**Figure 2:**
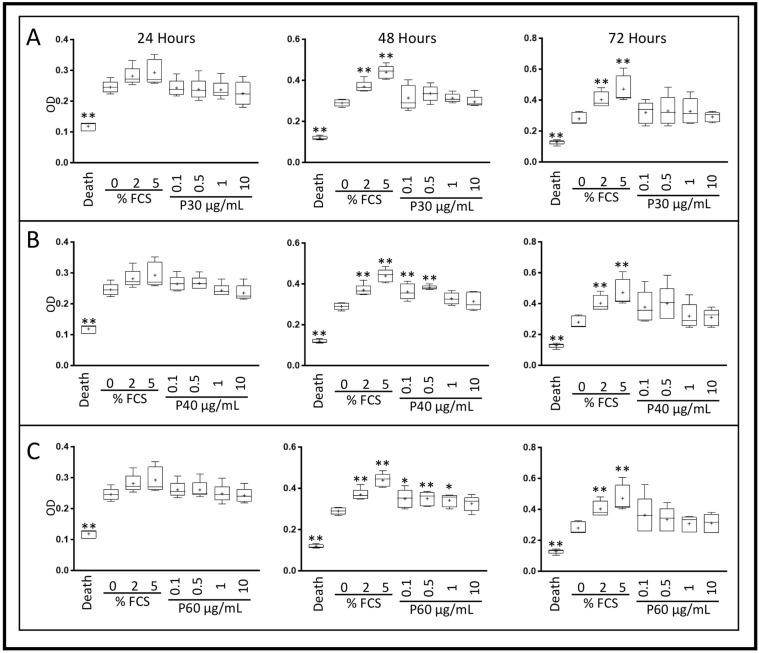
Effect of low concentrations of Promerim^®^30, 40, and 60 on the mitochondrial activity of equine articular chondrocytes cultured in the absence of serum. Equine articular chondrocytes were amplified and seeded at P3. At 80% of confluency, the cells were treated with Promerim^®^30 (**A**), 40 (**B**), 60 (**C**) at several concentrations (0.1, 0.5, 1, and 10 μg/mL) in the absence of FCS and then cultured for 24, 48 and 72 h in hypoxia. Controls with 0%, 2% and 5% of fetal calf serum (FCS), and a death control (triton-induced death) were included. The levels of formazan were measured (OD) in the media at the end of the incubation period (XTT kit, Roche). Data are represented as box plots (*n* = 5). Statistical analyses were performed using the Mann–Whitney test (* *p* < 0.05; ** *p* < 0.01) and the 0% FCS condition was used as a reference. P30, P40, and P60: Promerim^®^30, Promerim^®^40 and Promerim^®^60.

**Figure 3:**
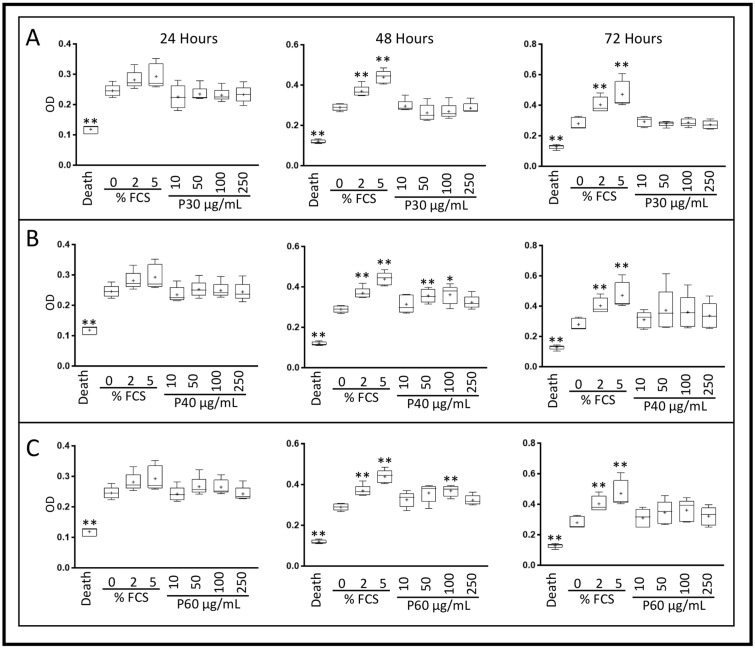
Effect of high concentrations of Promerim^®^30, 40, and 60 on the mitochondrial activity of equine articular chondrocytes cultured in the absence of fetal calf serum (FCS). Equine articular chondrocytes were amplified and seeded at P3. At 80% of confluency, the cells were treated with Promerim^®^30 (**A**), 40 (**B**), 60 (**C**) at several concentrations (10, 50, 100, and 250 μg/mL) in the absence of FCS and then cultured for 24, 48, and 72 h in hypoxia. Controls with 0%, 2% and 5% of FCS, and a death control (triton-induced death) were included. The levels of formazan were measured (OD) in the media after 24, 48 and 72 h of culture (XTT kit, Roche). Data are represented as box plots (*n* = 5). Statistical analyses were based on the Mann–Whitney test (* *p* < 0.05; ** *p* < 0.01) and the 0% FCS condition was used as a reference. P30, P40 and P60: Promerim^®^30, Promerim^®^40, and Promerim^®^60.

We then investigated the effect of Promerim^®^ on the mitochondrial metabolic activity of eACs (XTT assay) in serum-free media. In hypoxia, regardless of the duration of the incubation and the concentration of Promerim^®^30, eAC mitochondrial activity remained unchanged, compared with the control 0% FCS condition (Figure 2A and Figure 3A). On the other hand, Promerim^®^40 at 0.1 µg/mL, 0.5 µg/mL, 50 µg/mL, and 100 µg/mL for a 48 h incubation period led to a significant increase in the metabolic activity of eACs compared with the 0% FCS control (Figure 2B and Figure 3B).

After 48 h of treatment with Promerim^®^60 used at 0.1 µg/mL, 0.5 µg/mL, 1 µg/mL, and 100 µg/mL, eAC metabolic activity increased significantly, compared with the 0% FCS control. We observed the same trend at the other concentrations of Promerim^®^60 for 48 h or for all the concentrations when Promerim^®^60 was added for 72 h, although the results were not significant (Figure 2C and Figure 3C). We observed similar results when eACs were cultured in normoxia, especially for Promerim^®^40 and Promerim^®^60 at low and high concentrations after 48 h of incubation (Figures S2 and S3).

**Figure 4:**
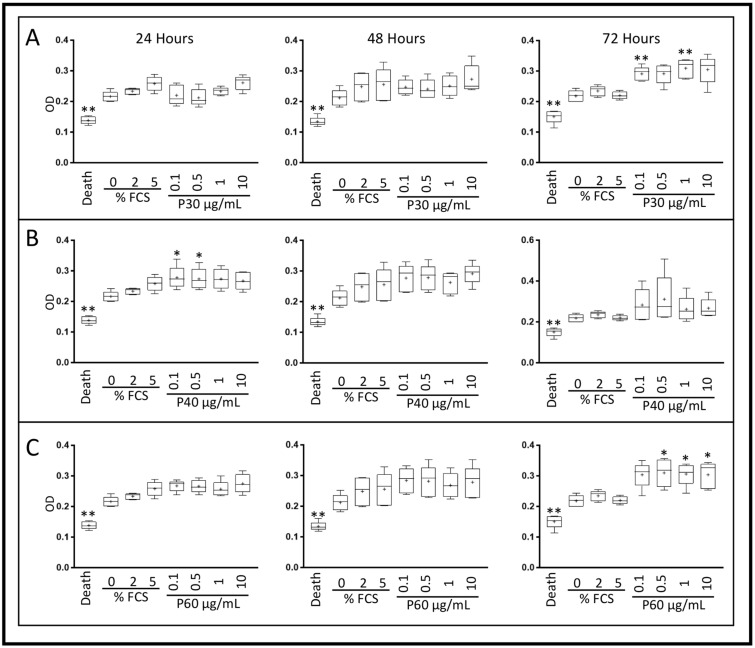
Effect of low concentrations of Promerim^®^30, 40, and 60 on the mitochondrial activity of equine articular chondrocytes cultured in the presence of 2% fetal calf serum (FCS). Equine articular chondrocytes were amplified and seeded at P3. At 80% of confluency, the cells were treated with Promerim^®^30 (**A**), 40 (**B**), 60 (**C**) at several concentrations (0.1, 0.5, 1, and 10 μg/mL) in the presence of 2% FCS and then cultured for 24, 48 and 72 h in hypoxia. Controls with 0%, 2% and 5% of FCS, and a death control (triton-induced death) were included. The levels of formazan were measured (OD) in the media at the end of the incubation period (XTT kit, Roche). Data are represented as box plots (*n* = 5). Statistical analyses were based on the Mann–Whitney test (* *p* < 0.05; ** *p* < 0.01) and the 0% FCS condition was used as a reference. P30, P40 and P60: Promerim^®^30, Promerim^®^40, and Promerim^®^60.

**Figure 5:**
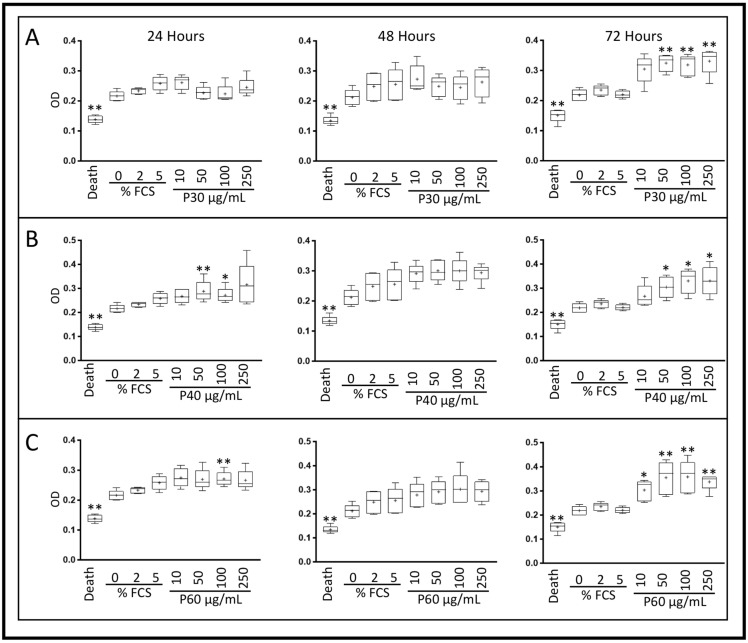
Effect of high concentrations of Promerim^®^30, 40, and 60 on the mitochondrial activity of equine articular chondrocytes cultured in the presence of 2% fetal calf serum (FCS). Equine articular chondrocytes were amplified and seeded at P3. At 80% of confluency, the cells were treated with Promerim^®^30 (**A**), 40 (**B**), 60 (**C**) at several concentrations (10, 50, 100, 250 μg/mL) in the presence of 2% of FCS and then cultured for 24, 48 and 72 h in hypoxia. Controls with 0%, 2% and 5% of FCS, and a death control (triton-induced death) were included. The levels of formazan were measured (OD) in the media at the end of the culture period (XTT kit, Roche). Data are represented as box plots (*n* = 5). Statistical analyses were based on the Mann–Whitney test (* *p* < 0.05; ** *p* < 0.01) and the 2% FCS condition was used as a reference. P30, P40 and P60: Promerim^®^30, Promerim^®^40, and Promerim^®^60.

**Figure 6:**
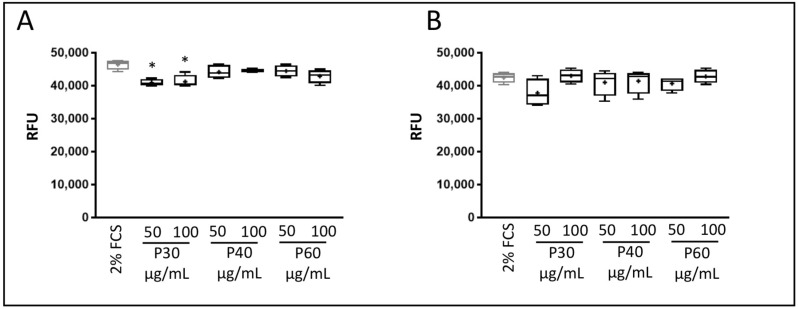
Effect of Promerim^®^30, 40, and 60 on the senescence of equine articular chondrocytes. Equine articular chondrocytes were amplified and seeded at P3. At 80% of confluency, the cells were treated with Promerim^®^30, 40, and 60 at two concentrations (50 and 100 μg/mL) in the presence of 2% fetal calf serum (FCS) and then cultured for 72 h in hypoxia (**A**) and normoxia (**B**). Controls with 2% FCS were performed. The levels of β-galactosidase were measured 3 days post-treatment. Data are represented as box plots (*n* = 4). Statistical analyses were based on the Mann–Whitney test (* *p* < 0.05) and the 2% FCS condition was used as a reference. P30, P40 and P60: Promerim^®^30, Promerim^®^40, and Promerim^®^60.

Because subsequent redifferentiation experiments were performed in the presence of 2% FCS, we also assessed the effects of Promerim^®^ on eACs (metabolic activity, senescence) cultured in presence of 2% FCS and in hypoxia (Figure 4, Figure 5 and Figure 6). The effect of normoxia was also evaluated (Figures S4 and S5).

In hypoxia, Promerim^®^30 increased eAC mitochondrial metabolic activity after 72 h of treatment, regardless of the concentration used, reaching levels similar to those observed with 5% FCS (Figure 4A and Figure 5A). Regardless of its concentration of use, Promerim^®^40 started to increase eAC mitochondrial metabolic activity as early as 24 h of treatment. This increase was confirmed when eACs were cultured for 72 h in the presence of Promerim^®^40. A trend was also observed for 48 h of incubation in the presence of Promerim^®^40 (Figure 4B and Figure 5B). Promerim^®^60, like Promerim^®^30, induced a significant increase in eAC metabolic activity when the incubation time lasted 72 h, although the same trend was observed for shorter incubation times (Figure 4C and Figure 5C).

When eACs were cultured in normoxia, the Promerim^®^ hydrolysates led to similar effects on eAC mitochondrial activity, although their kinetics of action changed slightly. We observed an increase in activity when eACs were cultured with Promerim^®^30 or Promerim^®^40 for 72 h and an increase as early as 24 h of incubation with Promerim^®^60 (Figures S4 and S5).

We then investigated the effect of Promerim^®^ hydrolysates on eAC senescence. To do so, eACs were treated with Promerim^®^30, 40, or 60 used at 50 and 100 μg/mL for 72 h, and in the presence of 2% FCS either under hypoxia or in normoxia. Then, we assessed SA-β-galactosidase activity. In hypoxia, Promerim^®^30 significantly downregulated SA-β-galactosidase activity compared with the 2% FCS control (Figure 6A). Although no significant differences were observed when eACs were treated with Promerim^®^40 or 60, the activity of SA-β-galactosidase also tended to decrease. In normoxia (Figure 6B), the activity of SA-β-galactosidase was not significantly modulated, compared with the 2% FCS control. However, Promerim^®^30 used at 50 μg/mL tended to decrease the activity of β-galactosidase. Altogether, these data show that Promerim^®^30, 40, and 60 are not cytotoxic for eACs; on the contrary, they favor eAC mitochondrial activity, and Promerim^®^30 inhibits cell senescence.

### 2.2. Effect of Promerim^®^ Hydrolysates on the Expression of mRNAs Encoding Characteristic Biomarkers of Chondrocytes

Then, we assessed the effect of Promerim^®^30 and 40 when chondrocytes were cultured as cartilaginous organoids. In this culture model, chondrocytes evolve in a microenvironment similar to an in vivo context. Furthermore, the cells were cultured in the presence of BMP-2 to maintain a chondrogenic environment, or in the presence of IL-1β to mimic the pro-inflammatory environment that occurs during OA. The combination of BMP-2 and IL-1β was used to mimic an early stage of OA. Organoids were cultured in the presence of Promerim^®^ hydrolysates at low concentrations (0.1 and 0.5 µg/mL) and at relatively high concentrations (50 and 100 µg/mL) for 7 days. The control condition corresponds to organoids cultured in the presence of the 3D medium alone.

**Figure 7:**
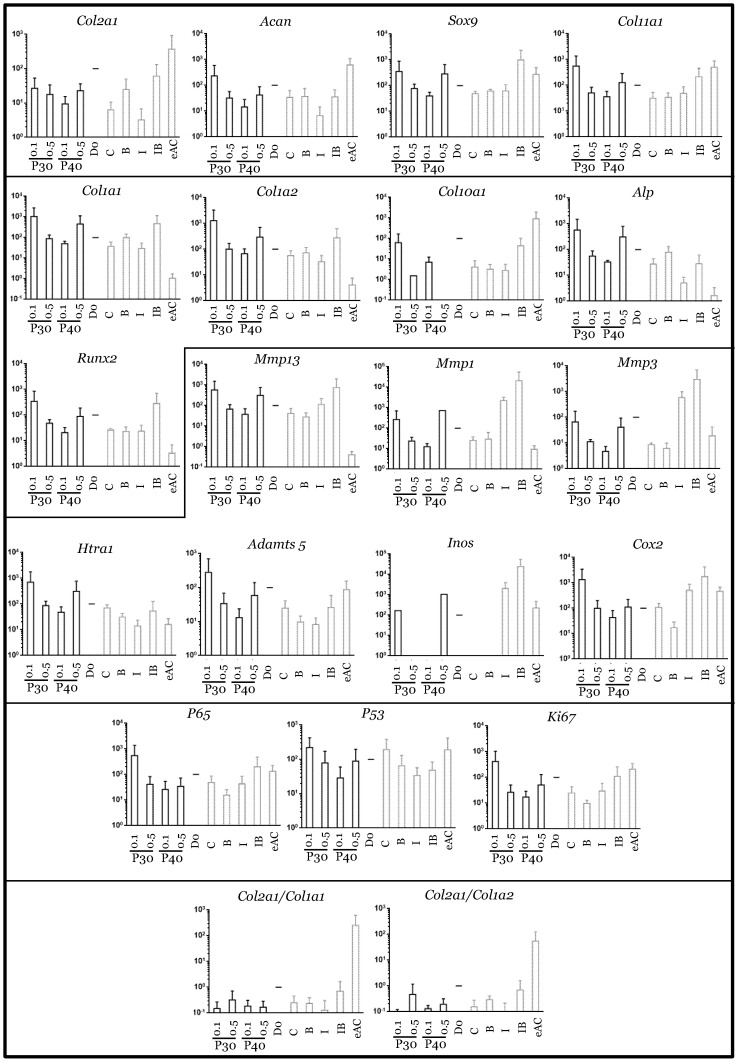
Comparison of mRNA expression in equine articular chondrocytes in the presence or absence of low concentrations of Promerim^®^30 and 40. Equine articular chondrocytes were grown in type I/III collagen sponges at the third passage (P3). They were incubated for 7 days in hypoxia in the absence (C: control) or presence of Promerim^®^30 or Promerim^®^40 (P30 and P40) (0.1 and 0.5 µg/mL), or BMP-2 (B), or IL-1 (I), or BMP-2 and IL-1 (IB). At the end of incubation period, their mRNA was extracted as described in Materials and Methods. The mRNAs were estimated using RT-qPCR after normalization with respect to the β-actin reference gene. Transcript expression is shown in arbitrary units. The *Col2a1*:*Col1a1* and *Col2a1*:*Col1a2* ratios are given. The results are shown as histograms and the significance of the values between the different treatments and the control (C) was tested using a Mann–Whitney test; *n* = 3. eAC: mRNA extracts obtained from equine articular chondrocytes released from cartilage after overnight enzymatic digestion were used as controls. D0: cells seeded in sponges and arrested after 16 h of incubation.

**Figure 8:**
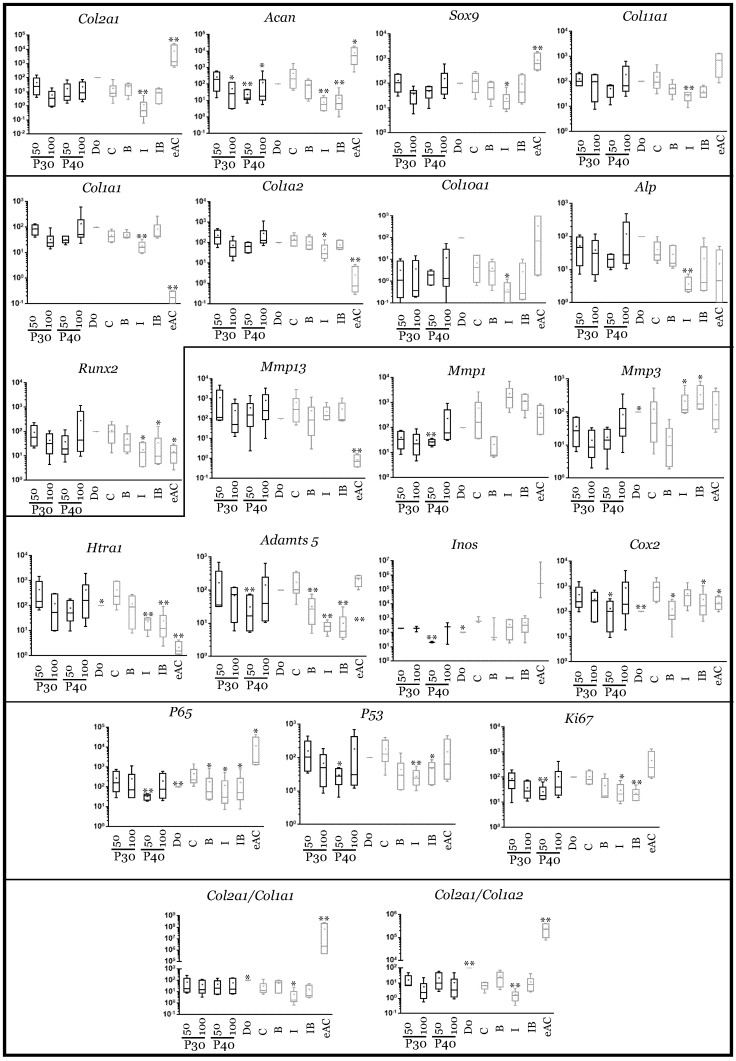
Comparison of mRNA expression in equine articular chondrocytes in the presence or absence of high concentrations of Promerim^®^30 and 40. Equine articular chondrocytes were grown in type I/III collagen sponges at the third passage (P3). They were incubated for 7 days in hypoxia in the absence (C: control) or presence of Promerim^®^30 or Promerim^®^40 (P30 and P40) (50 and 100 µg/mL), or BMP-2 (B), or IL-1 (I), or BMP-2 and IL-1 (IB). At the end of incubation period, their mRNA was extracted as described in Materials and Methods. The mRNAs were estimated using RT-qPCR after normalization with respect to the β-actin reference gene. Transcript expression is shown in arbitrary units. The *Col2a1*:*Col1a1* and *Col2a1*:*Col1a2* ratios are given. The results are shown as box plots (median, quartiles, extreme values) and the significance of the values between the different treatments and the control (C) was tested using a Mann–Whitney test (* *p* < 0.05; ** *p* < 0.01); *n* = 6. eAC: mRNA extracts obtained from equine articular chondrocytes released from cartilage after overnight enzymatic digestion were used as controls. D0: cells seeded in sponges and arrested after 16 h of incubation.

When Promerim^®^30 and 40 were used at 0.1 µg/mL and 0.5 µg/mL, respectively, the typical and atypical transcript levels of chondrocyte phenotypic markers both tended to increase (Figure 7). Promerim^®^30 at 0.5 µg/mL and Promerim^®^40 at 0.1 µg/mL had no effect on the steady-state amounts of the typical and atypical molecules. Thus, the *Col2a1*:*Col1a1* and *Col2a1*:*Col1a2* mRNA ratios remained unchanged compared with the control. Similar results were observed when Promerim^®^30 and 40 were used at 50 µg/mL and 100 µg/mL, except a decrease in *Acan* mRNA levels (Promerim^®^30 at 100 µg/mL, and Promerim^®^40 at both concentrations) (Figure 8). Interestingly, although Promerim^®^ hydrolysates used at low concentrations had no significant effect on *Mmp3*, *Mmp1*, or *Htra1* transcript levels, they led to a decrease in the steady-state amounts of *Mmp1* and *Mmp3* when they were used at 50 µg/mL and 100 µg/mL. The *Htra1* and *Adamts5* mRNA levels were downregulated only when Promerim^®^30 was used at 100 µg/mL, and when Promerim^®^40 was used at 50 µg/mL (Figure 7 and Figure 8). Only Promerim^®^40 tested at 50 µg/mL led to a decrease in mRNA levels of the proliferative marker *Ki67*, but also of *P53* and alinflammation-associated molecules (*Cox2*, *Inos*, and *P65*) (Figure 8).

**Figure 9:**
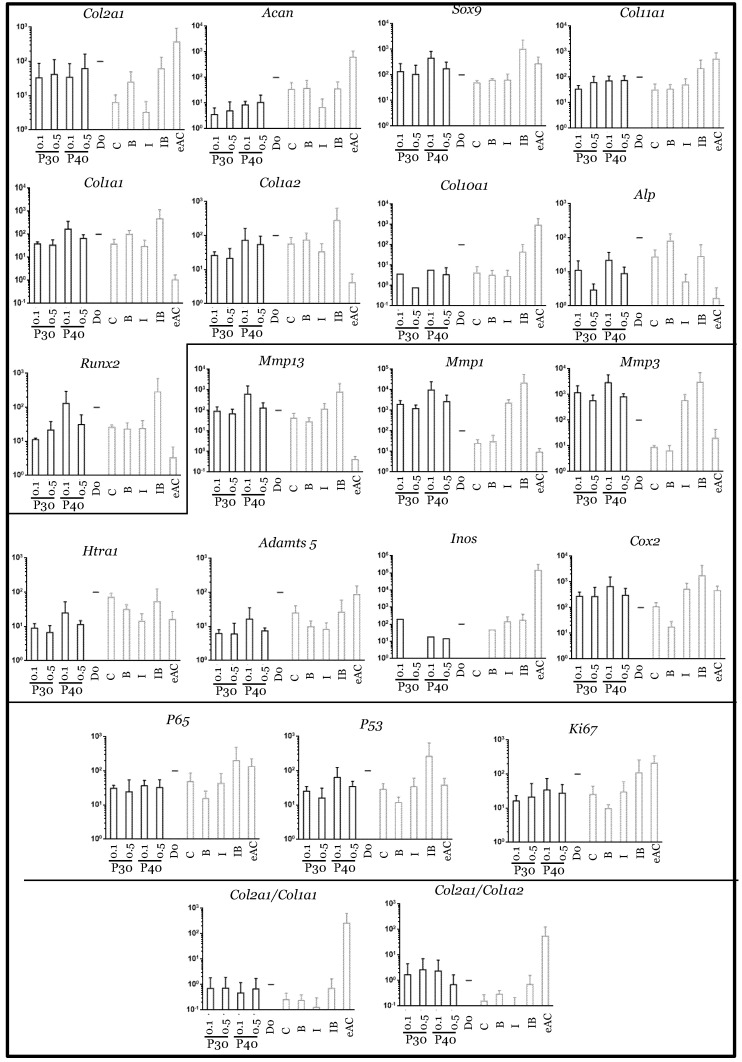
mRNA expression in equine articular chondrocytes treated with Promerim^®^30 and 40 used at low concentrations in the presence of IL-1. Equine articular chondrocytes were grown in type I/III collagen sponges at the third passage (P3). They were incubated for 7 days under hypoxia in the absence (C: control) or both the presence of IL-1 + P30 and IL-1 + P40, or BMP-2 alone (B), or IL-1 alone (I), or BMP-2 together with IL-1 (IB). The Promerim^®^ were used at the concentrations of 0.1 and 0.5 µg/mL. The mRNAs were estimated using RT-qPCR after normalization with respect to the β-actin reference gene. Transcript expression is shown in arbitrary units. The *Col2a1*:*Col1a1* and *Col2a1*:*Col1a2* ratios are given. The results are shown as histograms and the significance of the values between the different treatments and the control case (IL-1) was tested using a Mann–Whitney test; *n* = 3. eAC: mRNA extracts obtained from equine articular chondrocytes released from cartilage after overnight enzymatic digestion were used as controls. D0: cells seeded in sponges and arrested after 16 h of incubation. P30 and P40: Promerim^®^30 and 40.

**Figure 10:**
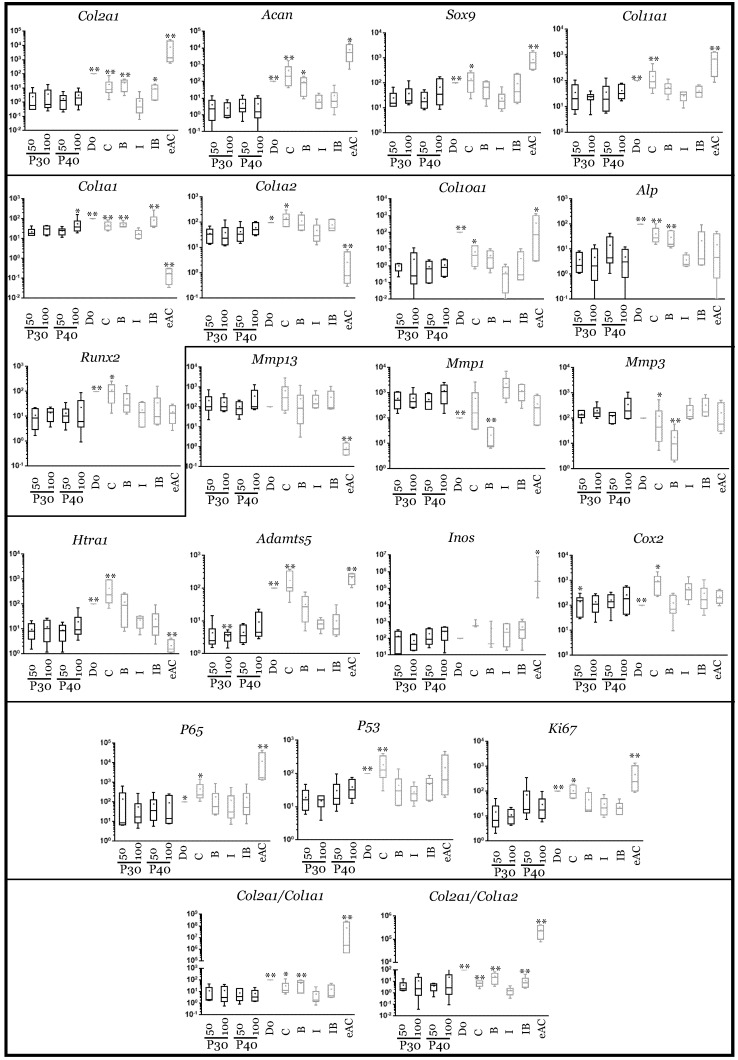
mRNA expression in equine articular chondrocytes treated with Promerim^®^30 and 40 used at high concentrations in the presence of IL-1. Equine articular chondrocytes were grown in type I/III collagen sponges at the third passage (P3). They were incubated during 7 days under hypoxia in the absence (C: control) or both the presence of IL-1 + P30 and IL-1 + P40, or BMP-2 alone (B), or IL-1 alone (I), or BMP-2 together with IL-1 (IB). The Promerim^®^ were used at the concentrations of 50 and 100 µg/mL. The mRNAs were estimated using RT-qPCR after normalization with respect to the β-actin reference gene. Transcript expression is shown in arbitrary units. The *Col2a1*:*Col1a1* and *Col2a1*:*Col1a2* ratios are given. The results are shown as box plots (median, quartiles, extreme values) and the significance of the values between the different treatments and the control case (IL-1) was tested using a Mann–Whitney test (* *p* < 0.05; ** *p* < 0.01); *n* = 6. eAC: mRNA extracts obtained from equine articular chondrocytes released from cartilage after overnight enzymatic digestion were used as controls. D0: cells seeded in sponges and arrested after 16 h of incubation. P30 and P40: Promerim^®^30 and 40.

In the presence of IL-1β, the use of Promerim^®^30 and 40 at 0.1 and 0.5 µg/mL led to an increase in *Col2a1* mRNA levels when compared to the IL-1ß treated samples, whereas the mRNA levels of the other phenotypic markers remained unchanged (Figure 9). Thus, the *Col2a1*:*Col1a1* and *Col2a1*:*Col1a2* ratios also increased. On the contrary, when both Promerim^®^ hydrolysate versions were tested at 50 and 100 µg/mL, the mRNA levels of the phenotypic markers remained unchanged compared with the eACs cultured in the presence of IL-1β only (Figure 10), even though *Col2a1* mRNA levels tended to increase slightly. The mRNA levels of proteases, as well as inflammatory markers, proliferative/senescence markers were not modulated by the addition of Promerim^®^ at 0.1 and 0.5 µg/mL (Figure 9). Although a statistically significant decrease of the mRNA steady-state amounts of *Adamts5* was observed only when Promerim^®^30 was used at 100 µg/mL, Promerim^®^30 (50 and 100 µg/mL) and 40 (50 µg/mL) tended to decrease the mRNA levels of the proteases *Htra1*, *Mmp1* and *Adamts5* (Figure 10). mRNA levels of *Cox2* also tended to be downregulated, although only Promerim^®^30 used at 50 µg/mL led to a statistically significant decrease.

**Figure 11:**
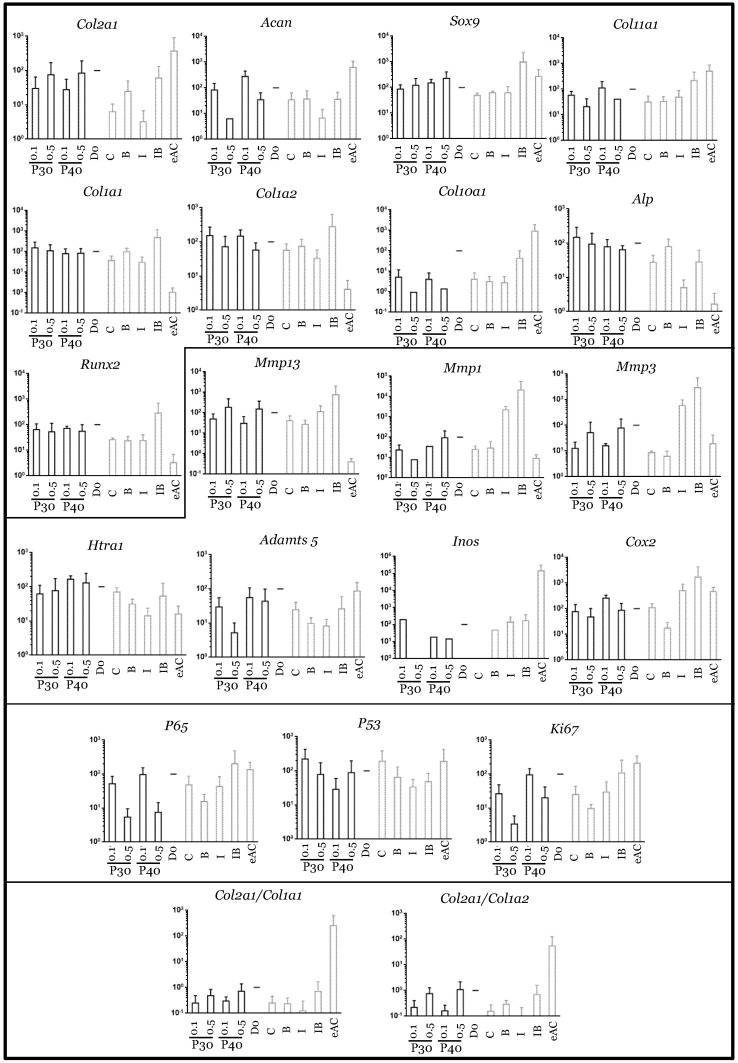
mRNA expression in equine articular chondrocytes treated with low concentrations of Promerim^®^30 and 40 in the presence of BMP-2. Equine articular chondrocytes were grown in type I/III collagen sponges at the third passage (P3). They were incubated during 7 days under hypoxia in the absence (C: control) or both the presence of BMP-2 + P30 and BMP-2 + P40, or BMP-2 alone (B), or IL-1 alone (I), or BMP-2 and IL-1 (IB). The Promerim^®^ were used at the concentrations of 0.1 and 0.5 µg/mL. The mRNAs were estimated using RT-qPCR after normalization with respect to the β-actin reference gene. Transcript expression is shown in arbitrary units. The *Col2a1*:*Col1a1* and *Col2a1*:*Col1a2* ratios are given. The results are shown as histograms and the significance of the values between the different treatments and the control case (BMP-2) was tested using a Mann–Whitney test; *n* = 3. eAC: mRNA extracts obtained from equine articular chondrocytes released from cartilage after overnight enzymatic digestion were used as controls. D0: cells seeded in sponges and arrested after 16 h of incubation. P30 and P40: Promerim^®^30 and 40.

Under chondrogenic differentiating conditions, in the presence of BMP-2, Promerim^®^30 and 40 led to a slight increase in *Col2a1* mRNA levels, only when they were added at 0.5 µg/mL when compared to the BMP-2 treated samples (Figure 11). The mRNA levels of the other phenotypic markers, and also of *Col1a1* and *Col1a2*, were unchanged upon the addition of the Promerim^®^30 or 40 used at 0.1, 50, and 100 µg/mL (Figure 11 and Figure S6). Thus, the ratios *Col2a1*:*Col1a1* and *Col2a1*:*Col1a2* tended to increase, compared with the BMP-2 control, only when Promerim^®^30 and 40 were used at 0.5 µg/mL. Promerim^®^30 and 40 (0.1 and 0.5 µg/mL) tended to increase the mRNA levels of the proteases *Mmp3*, *Adamts5*, and *Htra1* (for Promerim^®^30, only at 0.1 µg/mL for the latter). *Cox2* and *Ki67* mRNA amounts also increased. At concentrations of 50 µg/mL, only Promerim^®^40 led to an increase in *Mmp1*, *Adamts5*, *P65*, and *Cox2* (Figure S6). At 100 µg/mL, compared with the BMP-2 condition, neither Promerim^®^ led to any modulation of the mRNA levels of the proteases, and inflammation- and proliferation-associated molecules studied.

**Figure 12:**
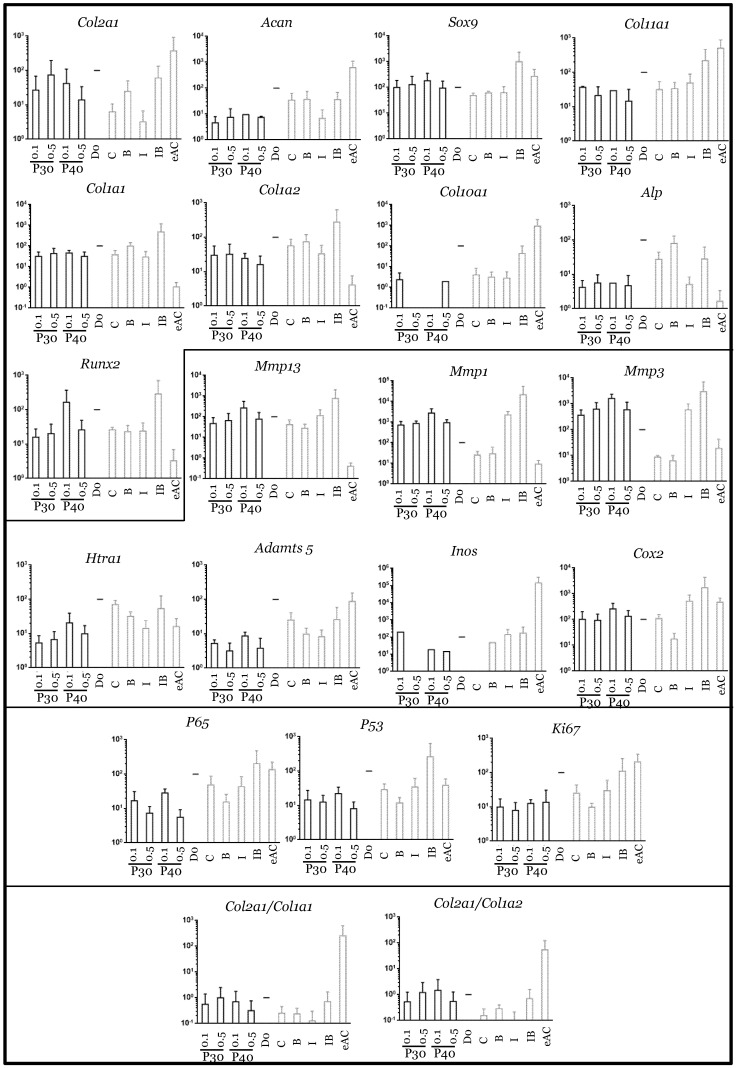
mRNA expression in equine articular chondrocytes treated with low concentrations of Promerim^®^30 and 40 in the presence of both IL-1 and BMP-2. Equine articular chondrocytes were grown in type I/III collagen sponges at the third passage (P3). They were incubated for 7 days under hypoxia in the absence (C: control) or both the presence of P30 + IL-1 + BMP-2 and P40 + IL-1 + BMP-2, or BMP-2 alone (B), or IL-1 alone (I), or BMP-2 together with IL-1 (IB). The Promerim^®^ were used at the concentrations of 0.1 and 0.5 µg/mL. The mRNAs were estimated using RT-qPCR after normalization with respect to the β-actin reference gene. Transcript expression is shown in arbitrary units. The *Col2a1*:*Col1a1* and *Col2a1*:*Col1a2* ratios are given. The results are shown as histograms and the significance of the values between the different treatments and the control case (IL-1 + BMP-2) was tested using a Mann–Whitney test; *n* = 3. eAC: mRNA extracts obtained from equine articular chondrocytes released from cartilage after overnight enzymatic digestion were used as controls. D0: cells seeded in sponges and arrested after 16 h of incubation. P30 and P40: Promerim^®^30 and 40.

Finally, in pre-arthritic conditions, in the presence of both IL-1β and BMP-2, Promerim^®^ hydrolysates at low concentrations (0.1 and 0.5 µg/mL) decreased the mRNA levels of most of the atypical phenotypic molecules studied, notably *Col1a1*, *Col1a2*, and those encoding the proteases, when compared to the IL-1β + BMP-2 control (Figure 12). Nevertheless, even though *Col2a1* mRNA levels were not modulated by Promerim^®^30 or 40, *Col11a1*, *Sox9*, and *Acan* mRNA levels tended to decrease. The mRNA levels of the proliferation-associated molecules ki67, but also p53, the inflammation-associated molecules Cox2 and p65, and the hypertrophic markers (*Col10a1*, *Mmp13*, *Runx2*, and *Alp*) decreased slightly. In contrast, when both Promerim^®^30 and 40 were added at the highest concentrations (50 and 100 µg/mL), all the effects detected at low Promerim^®^ concentrations were lost, because none of the mRNA of all the markers studied remained modulated (Figure S7).

### 2.3. Effect of Promerim^®^ Hydrolysates on the Protein Expression of Type II and I Collagens and Htra1

**Figure 13:**
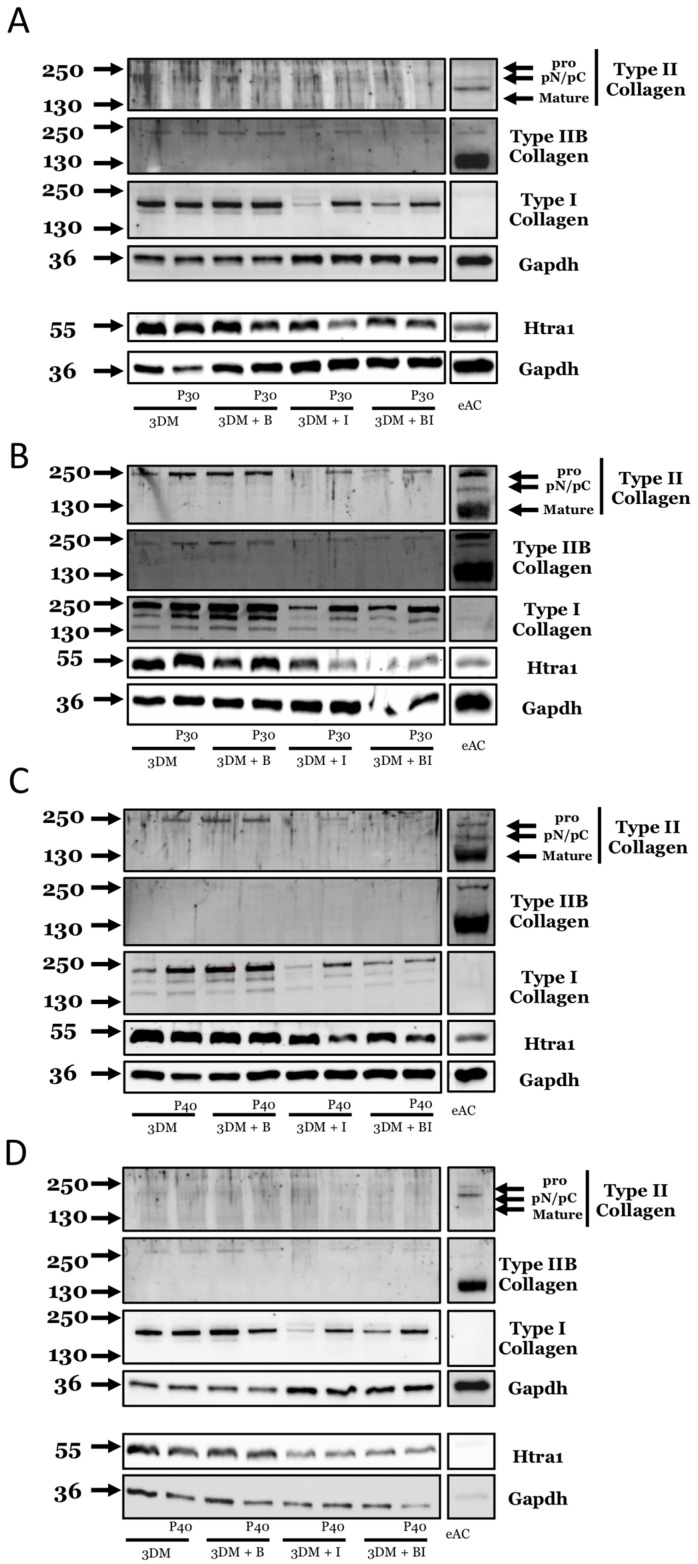
Effect of low concentrations of Promerim^®^ on chondrocyte protein expression of types II and I collagens and Htra1. Equine articular chondrocytes (eACs) at P3 were inoculated in collagen sponges with different treatments: 3D control culture medium (3DM), B (BMP-2), I (IL-1), B + I (BMP-2 + IL-1), P30 (Promerim^®^30), P40 (Promerim^®^40). (**A**) P30 at 0.1 µg/mL; (**B**) P30 at 0.5 µg/mL; (**C**) P40 at 0.1 µg/mL; (**D**) P40 at 0.5 µg/mL. Protein expression of equine articular chondrocytes was evaluated after 7 days of 3D culture in the presence and absence of treatment. The total protein extracts were separated by electrophoresis in 10% acrylamide gel in denaturing condition. The molecular weights expressed in kDa are shown on the left part of the panels and the antibodies used on the right part of the panels. Representative blots from different eAC strains are shown (at least *n* = 3). eAC: protein extracts obtained from equine articular cartilage are used as controls.

**Figure 14:**
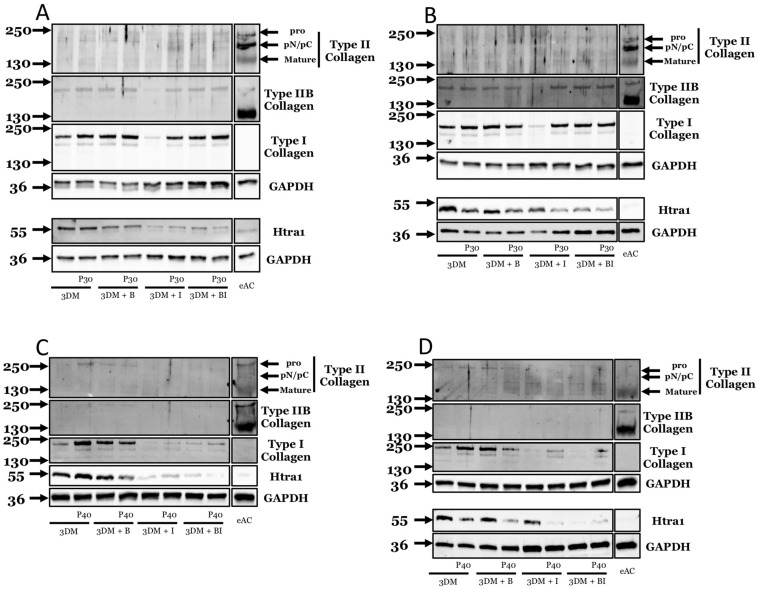
Effect of high concentrations of Promerim^®^ on chondrocyte protein expression of types II and I collagens and Htra1. Equine articular chondrocytes (eACs) at P3 were inoculated in collagen sponges with different treatments: 3D control culture medium (3DM), B (BMP-2), I (IL-1), B + I (BMP-2 + IL-1), P30 (Promerim^®^30), P40 (Promerim^®^40). (**A**) P30 at 50 µg/mL; (**B**) P30 at 100 µg/mL; (**C**) P40 at 50 µg/mL; (**D**) P40 at 100 µg/mL. Protein expression of equine articular chondrocytes was evaluated after 7 days of 3D culture in the presence and absence of treatment. The total protein extracts were separated by electrophoresis in 10% acrylamide gel in denaturing condition. The molecular weights expressed in kDa are shown on the left part of the panels and the antibodies used on the right part of the panels. Representative blots from different eAC strains are shown (at least *n* = 3). eAC: protein extracts obtained from equine articular cartilage are used as controls.

We then assessed the quality of the neo-synthesized extracellular matrix of the organoids by monitoring the protein levels of type IIB, II, and I collagens, as well as the serine protease Htra1. We used eACs at P3 to better observe a possible increase in type II collagen isoforms, because eACs cultured in control conditions produced a very low amount of type II collagen, as previously reported [31]. Promerim^®^30 did not show a striking increase in the synthesis of the type II collagen isoforms, regardless of its concentration (Figure 13A,B and Figure 14A,B). Nevertheless, in the presence of IL-1β, Promerim^®^30 systematically led to a slight increase in type IIB collagen expression, compared with the IL-1β treatment. Additionally, without BMP-2 or IL-1β, Promerim^®^30 (0.5, 50, and 100 µg/mL) led to an increase in type I collagen protein amounts (Figure 13B and Figure 14A,B). Although in the presence of BMP-2, Promerim^®^30 had no obvious effect, it was able to systematically counteract the decrease of the type I collagen protein amounts observed in the presence of IL-1β. In combination with both BMP-2 and IL-1β, Promerim^®^30 (0.1, 0.5, and 50 µg/mL) also increased type I collagen protein levels. IL-1β practically systematically decreased Htra1 expression (Figure 13A–C and Figure 14A–D). The inflammatory conditions mimicked by incubation in the presence of IL-1β favored the effect of the Promerim^®^30 on the downregulation of Htra1 expression.

Altogether, these data suggest that Promerim^®^30 holds promise for enhanced extracellular matrix accumulation in an inflammatory microenvironment.

Regarding Promerim^®^40 at 0.1 µg/mL, used alone or in combination with IL-1β, type IIB collagen protein levels remained unchanged and expression was very weak. Type I collagen protein amounts increased when Promerim^®^40 was used alone or in combination with IL-1β. Htra1 protein levels tended to decrease when Promerim^®^40 was combined with IL-1β or with the combination of both IL-1β and BMP-2, compared with the respective controls.

When Promerim^®^40 was used at 0.5 µg/mL, we did not observe any variation in type II collagen or Htra1 protein amounts (Figure 13D). Type I collagen synthesis was increased when Promerim^®^40 was concomitantly added with IL-1β alone or in association with BMP-2.

Promerim^®^40 used at 50 µg/mL or 100 µg/mL did not seem to modulate type II collagen expression (Figure 14C,D). On the other hand, when used alone, Promerim^®^40 at 50 µg/mL strongly increased type I collagen expression. Finally, Promerim^®^40 led to a decrease in Htra1 protein amount in the presence IL-1β.

Promerim^®^40 used at 100 µg/mL, alone or in combination with IL-1β or IL-1β + BMP-2, upregulated the type I collagen accumulation (Figure 14D). On the other hand, a decrease in type I collagen synthesis was observed when Promerim^®^40 was combined with BMP-2.

### 2.4. Promerim^®^30 and 40 Promote Proliferation, Whereas Promerim^®^60 Promotes Migration of eACs

**Figure 15:**
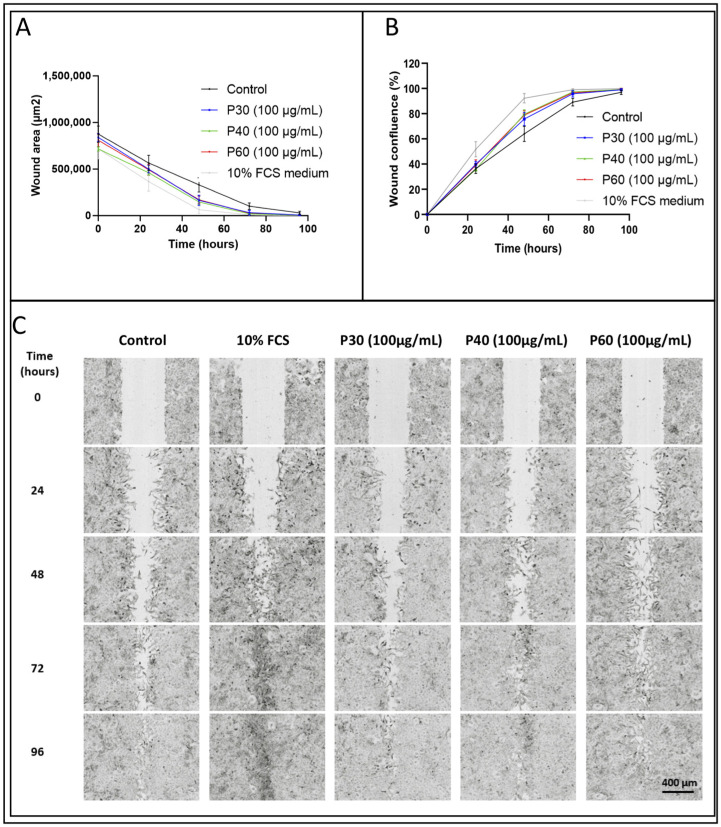
Wound-filling analysis following a scratch wound assay. Equine articular chondrocytes (eACs) were seeded at P2. At 90% of confluency, a scratch wound assay was performed using a WoundMaker^TM^ kit (Essen BioScience) and then the treatments were added. eACs were incubated in the presence of a culture medium containing 2% fetal calf serum (FCS) (Control), and P30, P40, or P60 at 100 µg/mL. The cells were also incubated in the presence of 10% FCS. The wound areas (**A**) were monitored until 96 h of incubation, and the wound confluences (**B**) were calculated using the ImageJ software. Every 24 h, photographs (**C**) of the wound were taken with Incucyte. The significance of the values between the different treatments and the control case was tested using a two-way ANOVA followed by Bonferroni test for multiple comparisons (*n* = 4). P30, P40 and P60: Promerim^®^30, Promerim^®^40, and Promerim^®^60.

Enhancing proliferation of eACs and promoting the migration of eAC/progenitors to the chondral lesion areas is of particular interest to try to delay OA outcomes. The effects of Promerim^®^30, 40, and 60 on the proliferation/migration of eACs was assessed using a wound healing assay. At 48 h post-scratching, eACs treated with Promerim^®^40 and 60 colonized the wound areas to a higher extent compared with eACs cultured with the control medium containing 2% FCS (control) (Figure 15A). Thus, the cell confluence in the wound area increased significantly when eACs were treated with Promerim^®^40 and 60 (Figure 15B). Although the results were not statistically significant, the same trend was observed when eACs were cultured with Promerim^®^30. Whereas the increase in the confluence when eACs were incubated in the presence of Promerim^®^30 and 40 seemed to be due to an increase in cell proliferation, Promerim^®^60 seemed to favor migration, because we observed spindle-shaped eACs, characteristic of migrating cells (Figure 15C).

## 3. Discussion

Numerous nutraceuticals have been studied to relieve discomfort associated with articular disorders, including OA [17,32]. There are hundreds of food supplements available on the market to treat OA. Among them are the most frequently used active ingredients, such as collagen hydrolysates [33], *Curcuma longa* and curcumin extracts [34,35], and *Boswellia serrata* [36,37], which have demonstrated highly and clinically significant short-term reduction in pain [38]. Others, such as undenatured type II collagen [39], avocado and unsaponifiable soy [40], glucosamine, and chondroitin have shown statistically significant improvements in pain management [22,41,42], but their clinical significance has not been clearly identified, because long-term effects on inflammation and matrix regeneration still remain to be demonstrated [38]. Studies have also shown that, in women suffering from moderate knee OA, daily oral intake of collagen peptides for 6 months reduces pain and increases the mobility of the affected joint [25]. Additionally, McAlindon and collaborators performed double-blind clinical trials on 29 individuals aged 49 years and older with severe to moderate OA of the knee. Patients were treated with collagen hydrolysates versus placebo and showed an increase in the quality of the ECM quality after 24 weeks of treatment [15]. These results are in agreement with those we observed in vitro and confirm that the use of collagen hydrolysates in the treatment of OA is relevant. 

Collagen hydrolysates belong to one of the most interesting class of nutraceuticals to improve OA condition or delay its outcomes, especially through the downregulation of the inflammation and degradation molecules [28]. The use of collagen hydrolysates in vivo dates back to a 2000 study that reported that collagen hydrolysates were able to reduce pain in patients with knee or hip OA, and increase the blood concentration of hydroxyproline [43]. Since then, different sources of collagens have been studied. For example, chicken collagen hydrolysates keep inflammation and ECM degradation in check in mice, and improve joint mobility in humans [44,45]. Likewise, shark collagen hydrolysates can act on whole joint tissues to counteract OA outcomes in rabbits [46]. Collagen hydrolysates used in our present study were derived from enzymatic hydrolysis into peptides that are more easily absorbed and have a strong affinity with water [47]. The marine environment is very specific—fish evolve in cold, salty environments, and can be subjected to high pressures. These elements differ from collagens derived from farmed land animals (cattle, pigs) and give them different physico-chemical properties due to their amino acid composition. Collagens of marine origin have antibacterial, antioxidant, neuroprotective, and anti-aging properties [48,49,50,51]. In addition, marine peptides are believed to promote skin healing in rats [52]. Further in vitro studies to assess the effect of these hydrolysates on cartilage matrices remain to be performed. In this study, we studied the effect of three collagen hydrolysates of marine origin (Promerim^®^30 and Promerim^®^60 from fish skin, and Promerim^®^40 from fish cartilage) on the metabolic activity, senescence, and phenotype of eACs, and on the subsequent quality of cartilaginous organoids.

We first showed that Promerim^®^ hydrolysates are not cytotoxic for eACs. Cell viability tests also allowed us to choose four preferential Promerim^®^ concentrations to investigate their effects on eAC phenotypes. Promerim^®^ concentrations of 0.1 and 0.5 µg/mL were sufficient to obtain an increase in eAC cell metabolic activity. We also selected higher concentrations (X500; X1000) to assess whether the effects of Promerim^®^ depended directly on their concentrations, which was an essential question because Promerim^®^ hydrolysates are intended to be taken by mouth as a dietary supplement. During digestion, oligopeptides are further metabolized into bioactive di- and tri-peptides in the gastrointestinal tract, and are then released into the bloodstream [53]. Although it is well-known that peptides from dietary supplements can be found in the bloodstream, the concentration of the dietary supplement that actually circulates in the body is unknown. For example, upon oral ingestion of gelatin hydrolysates derived from porcine skin, chicken feet, and cartilage after 12 h of fasting, the peptide form of hydroxyproline increases significantly and reaches a maximum level (20–60 nmol/mL of plasma) after 1–2 h, and then decreases to half the maximum level 4 h after ingestion [48]. The main constituents of food collagen peptides in human serum and plasma have been identified as Pro-Hyp. In addition, small but significant amounts of Ala-Hyp, Ala-Hyp-Gly, Pro-Hyp-Gly, Leu-Hyp, Ile-Hyp, and Phe-Hyp have been detected [54]. Here, we demonstrated that regardless of their tested concentration, both Promerim^®^ hydrolysates were able to increase collagen production and metabolism activity, and decrease catabolism at the mRNA level (see below). Thus, the effect of Promerim^®^ is maintained in a large range of concentrations, which makes them relevant as food supplements.

OA is an evolutive disease characterized by sequential events. Following an initiating event, intra-articular inflammation occurs. Then, as a first response, chondrocytes increase their proliferation and ECM production composed of atypical collagen molecules, notably type I collagen. Finally, chondrocytes become senescent, undergo apoptosis, and cartilage ECM is inevitably degraded. These events accompany an imbalance in anabolism and catabolism, with an upregulation of several matrix metalloproteinases, aggrecanases, and serine proteases. Among serine proteases, Htra1 is upregulated in synovial fluid and articular cartilage in patients with OA and rheumatoid arthritis. *Htra1* mRNA levels can increase seven-fold in OA cartilage compared with healthy cartilage [55]. Htra1 plays a pivotal role in the emergence of OA because it degrades directly and indirectly, particularly via the upregulation of metalloproteinases, ECM molecules, and inhibits anabolic signaling by antagonizing the receptor of the TGF-β family [56]. Here, we demonstrated for the first time that the pro-osteoarthritic IL-1β downregulates Htra1 in articular chondrocytes. However, another cytokine, IFN-γ, has been reported to significantly inhibit basal and LPS-induced Htra1 expression in macrophages and fibroblasts, two cell types mainly involved in Htra1 synthesis in rheumatoid arthritis, through activation of the p38 MAPK pathway and subsequent activation and binding of STAT1 to the *Htra1* promoter [57].

Promerim^®^ hydrolysates seem to have promising effects to prevent or delay, at least in part, OA pathogenesis. They antagonize the initial steps of OA pathogenesis by decreasing inflammation and inhibiting proteases. Promerim^®^ hydrolysates were able to decrease the expression of *Cox2*, as well as proteases such as *Mmp-1*, *-3*, *Adamts5*, and *Htra1*, confirming previous reports. For example, collagen hydrolysates, associated with curcuminoid extracts, exhibit anti-inflammatory properties when added to in vitro cultures of bovine and human chondrocytes [58]. Other dietary supplements, such as methionine, unsaponifiable extracts of avocado, soybean, and plant flavonoids and bioflavonoids also have anti-inflammatory properties, notably through the modulation of the NFkappaB or oxidative stress pathways [59,60,61,62]. Many dietary supplements have been described as acting on the EPA/DHA (eicosapentaenoic acid/docosahexaenoic acid) pathway, leading to the synthesis of prostaglandin E_2_ (PGE_2_) and LT4 pro-inflammatory factors, such as fish oil, ginger, or devil’s claw (*Harpagophytum procumbens*) [63,64,65]. Various supplements are also described as simply playing an antioxidant role, such as glycosaminoglycans, frankincense, plant flavonoids and bioflavonoids [66,67,68,69,70]. On the contrary, we had previously shown that shell extracts of marine origin promote the catabolic pathway of human dermal fibroblasts, or the activity of MMP-1 in chondrocytes [71,72]. Nevertheless, the composition of the Promerim^®^ is very different from shell extracts—it does not contain high concentrations of marine minerals. We assume that the effects of Promerim^®^, at least in part, derive from matrikines. Indeed, the hydrolysates tested here are of marine origin. During specific enzymatic digestion, protein fragments are released and they can then be the source of small bioactive peptides, also called matrikines [73], that may be the main players that make dietary supplements promising sources in rheumatology and for the treatment of OA to favor chondral defect healing. For example, some matrikines have been described as playing a role in wound healing and in the synthesis of anabolic components of fibroblasts [24]. In particular, the GHK tripeptide present in types I, V, and XI collagen is known to stimulate collagen and proteoglycan synthesis [23]. Altogether, our data demonstrate a promising role of Promerim^®^ in the prevention of the initial events triggering OA. In fact, OA is a degenerative pathology that most often evolves from micro-lesions. The results obtained in the present study prompted us to believe that hydrolysates could have effects at different stages of OA, such as in the earliest stages of OA by limiting the loss of chondral substance, limiting degradation by proteases, and promoting collagen syntheses. Promerim^®^ could also have an effect when the microenvironment becomes inflammatory, where the articular cartilage suffers from deleterious damage. We showed that when chondrocytes evolve in a pro-inflammatory environment (through addition of IL-1), marine collagen hydrolysates may prevent the dedifferentiation of equine articular chondrocytes, limiting their senescence, and they also ensure the maintenance of ECM synthesis. Thus, Promerim^®^ favors collagen production and metabolism, especially in an inflammatory environment, and these collagen hydrolysates may therefore delay OA progression throughout the entire pathological process, from the earliest to the latest stages. Previous studies have also demonstrated an increase in the synthesis of type II collagens or proteoglycans when collagen hydrolysates are added to bovine chondrocytes (derived from bovine skin) that were cultured in vitro [74,75]. Other supplements have demonstrated an increase in the synthesis of proteins characteristic of hyaline cartilage. These include glycosaminoglycans [76], olive oil [77], or unsaponifiable extracts of avocado and soybean [78]. Other products of marine origin can stimulate the synthesis of type I and III collagens, as well as that of sulfated glycosaminoglycans (GAGs) on human fibroblasts [79].

The increase in ECM production (especially collagens) induced by Promerim^®^30 and 40 in control and pro-inflammatory conditions, as observed on Western blots, can be attributed to a decrease in the expression and activity of several proteases. Nevertheless, in some conditions, Promerim^®^ hydrolysates also upregulated the mRNA steady-state amounts of ECM molecules, notably the collagens. Thus, in addition to inhibiting catabolism, Promerim^®^ favors anabolism. The mechanism of action of the upregulation of anabolism remains to be elucidated, even though the downregulation of Htra1 may be partly responsible. In particular, Htra1 plays a double role—on the one hand, it degrades the ECM and on the other hand, it degrades the receptors of the TGF-β family [56]. TGF-β signaling pathways play a major role in maintaining chondrocyte differentiation [80,81]. This increase in Htra1 expression detected upon the development of OA is thought to be due to the activation of discoidin domain receptor-2 (DDr-2), which is a cell surface receptor that, when negatively regulated, leads to an increase in ECM degradation [82,83]. Further studies could be carried out to determine whether the Promerim^®^ has an effect on these receptors.

Chondrocyte senescence is a major event in OA pathogenesis, which precedes the later stages of OA, and subchondral bone exposure in particular. Senescence can be induced by a range of factors, such as age, being overweight, mechanical or oxidative stress, dedifferentiation, or apoptotic phenomena [84,85,86,87,88]. Studies have shown that the suppression of senescent chondrocytes delays the progression of induced OA in mice [89]. Thus, Promerim^®^30 may help delay OA evolution because it is able to inhibit eAC senescence.

Because we had already demonstrated the ability of marine-origin products to slightly enhance cell migration and in vitro repair [79], we addressed the question of whether Promerim^®^ hydrolysates were able to promote eAC migration, which is of in vivo interest. Thus, we carried out a wound-healing assay when eAC were cultured in the presence of Promerim^®^. Even though these results will require further investigation to define the mode of action of Promerim^®^, the increase in confluence in the wound area when eACs were cultured in presence of Promerim^®^30 and 40 seemed to be due to an increase in cell proliferation. This increase is consistent with the increase in metabolic activity—related in part to proliferation—observed in the XTT assays. On the contrary, Promerim^®^60 seemed to favor migration, because we observed spindle-shaped eACs, characteristic of migrating cells. Therefore, Promerim^®^ 60 may favor migration of the chondrocytes to the chondral defect, and eventually enhance cartilage synthesis to fill the defect, whereas Promerim^®^30 and 40 may be useful for enhancing the proliferation and the reservoir of chondrocytes located near the cartilage defect.

To reconstitute cartilage of hyaline quality, cartilage cell-based therapies using chondrocytes or mesenchymal stem cells (MSCs) derived from several tissues have been developed [9,90]. These techniques were initially based on the autologous chondrocyte implantation (ACI) procedure developed by Brittberg and colleagues [91]. Basically, these approaches can be divided into two steps: amplification and subsequent differentiation/redifferentiation of the cells. Over time, these techniques have been improved in several aspects (use of chondrogenic 3D biomaterials, hypoxia, cell sourcing, etc.). Several cell sources have been used, such as chondrocytes or stem cells from various adult/neonatal tissues, as well as several differentiation protocols [30,31,92]. Nevertheless, despite the high quality of the in vitro synthesized cartilage substitute, they are not strictly identical to in vivo healthy hyaline cartilage. Thus, differentiation strategies can be improved. In this context, Promerim^®^ represents a good candidate to foster hyaline-like ECM production. Western blot experiments revealed that both Promerim^®^30 and 40 increase collagen biosynthesis in control conditions, demonstrating that they could be chondrogenic, such as BMP-2. Interestingly, both Promerim^®^30 and 40 systematically counteracted the inhibitory effects of IL-1β on collagen protein amounts, with levels at least reaching those observed in control conditions and even those of eAC cultures treated with pro-chondrogenic BMP-2. Therefore, Promerim^®^ may be useful during the two phases of the ACI procedure to increase the proliferation/amplification of cells and foster their synthesis during the differentiation/redifferentiation step, while inhibiting their senescence (Promerim^®^ 30). Furthermore, collagen hydrolysates may be of interest to enhance MSC chondrogenesis, all the more so if these hydrolysates are as active as some of the members of the TGF-β family [92,93,94,95,96,97].

We showed here that Promerim^®^ is not cytotoxic for eACs, but enhances their metabolic activity, even inhibiting their senescence (Promerim^®^30 only). The Promerim^®^ hydrolysates also favored ECM accumulation in organoids, increasing collagen production while possibly decreasing its catabolism. Interestingly, the beneficial effects of Promerim^®^ were enhanced under pro-inflammatory conditions. This study is the first to demonstrate the beneficial effects of marine collagen hydrolysates to counteract each step of the OA pathogenesis. Hence, in vivo, Promerim^®^ may enhance the first response of chondrocytes to chondral injuries and further delay OA progression. Furthermore, because Promerim^®^ downregulates several proteases, ECM degradation may also be delayed.

## 4. Materials and Methods

### 4.1. Collagen Hydrolysates

Promerim^®^ peptides are produced by enzymatic hydrolysis characterized by the control of hydrolysis time, acidity (pH), and temperature. There is no chemical process of hydrolysis. Promerim^®^ are hydrolysates of fish skin and cartilage proteins composed of collagen peptides. These are oligopeptides, that is, small peptides characterized by low molecular weights below 1500 Dalton. They are composed of 2, to a maximum of 15 amino acids. The minimum amount of hydroxyproline is 5 g/100 g and the two major amino acids are proline (9 g/100 g) and glycine (21 g/100 g).

### 4.2. Isolation and Cell Culture

Equine articular chondrocytes (eAC) were isolated from cartilage biopsy of healthy equine metacarpal joint from adult horses (4–10 years). Chondrocytes derived from 12 different horses were used in this study. All procedures described in the present study were approved by the Ethics Committee for Animal Experimentation (ComEth ANSES/ENVA/UPEC, 94 701 Maisons-Alfort, France; n° 15-023 (10 March 2015), n° 10-0051 (10 September 2014). Healthy equine cartilage was cut into small slices, then sequentially incubated with type XIV protease *Streptomyces griseus* (2 mg/mL) (Sigma-Aldrich, Saint-Louis, MO, USA) for 45 min at 37 °C and type I collagenase from *Clostridium histolyticum* (2 mg/mL) (Invitrogen Life Technologies, Carlsbad, CA, USA) overnight, at 37 °C. The suspension was filtered through a 70 µm mesh nylon membrane and centrifuged, and eAC were counted in the presence of trypan blue to assess their viability. eAC were seeded in plastic flasks at a density of 2 × 10^4^ cells/cm^2^ and cultured in DMEM-high glucose (HG, 4.5 g/L BioWest, Nuaillé, France) supplemented with 10% of fetal calf serum (FCS) (Eurobio Scientific, Courtaboeuf, France). In addition, antibiotics and antifungal were added in all the media used (100 IU/mL of penicillin, 100 µg/mL of erythromycin, and 0.25 mg/mL of fungizone (Eurobio Scientific, Courtaboeuf, France).

### 4.3. XTT Assay

After the third passage (P3), eAC were seeded in a 96-well plate at a density of 2 × 10^4^ cells/cm^2^, then cultured with DMEM HG with 10% FCS for 72 h (37 °C, 5% CO_2_) to allow the cells to adhere and reach 80% confluence. The cells were then treated with different concentrations (0.1, 0.5, 1, 10, 50, 100, and 250 μg/mL) of Promerim^®^30, 40, or 60. The Promerim^®^ were diluted in series either in DMEM HG supplemented with 2% FCS or in serum-free DMEM HG (0% FCS). As controls, eAC were also cultured without Promerim^®^ in DMEM HG in the absence of serum or supplemented with 2% or 5% FCS. Experiments were performed under normoxia (21% O_2_) or in chondrocyte physioxic conditions in hypoxia at 3% O_2_. Cell metabolism activity was assessed 24 h, 48 h, or 72 h after the addition of the treatments. At the end of each culture period, XTT assay was performed according to the manufacturer’s instructions (Roche, Bale, Switzerland). Optical density (OD) measurements were made at 490 nm, and the background (690 nm) was subtracted. Measurements were made with a microplate reader (Spark control Magellan, TECAN^®^). All experiments were performed in triplicate, and are presented as the mean of five experiments.

### 4.4. Toxilight Assay

eAC were cultured under the same conditions as for the XTT assays. After 72 h of treatment, for each condition, 80 μL of the culture medium was transferred to a 96-well plate. One hundred μL of AK Reagent working solution (Interchim^®^ Bioluminescence Cytotoxicity Assay Kit, Interchim, Montluçon, France) were added in each well and incubated for 5 min at room temperature. The bioluminescence was then measured by a microplate reader (TECAN^®^). All experiments were carried out in triplicate and are presented as the mean of five experiments.

### 4.5. β-Galactosidase Activity

β-galactosidase activity was measured using the Cell Biolabs Cell Senescence Assay Kit (Cell Biolabs, San Diego, CA, USA). eAC were seeded at 2 × 10^4^ cells/cm^2^ in the presence of DMEM HG (2% FCS). At 80% of confluency, the Promerim^®^30, 40, or 60 were added (50 or 100 μg/mL, in normoxia or hypoxia). Then, the media were removed, eAC were washed with PBS 1X. The 1X cell lysis buffer containing PMSF (5 μL/mL) was added to the eAC. The lysates were recovered and centrifuged at 300 g (10 min, 4 °C). The supernatants were then transferred to a 96-well plate and incubated (16 h, 37 °C) in the presence of the substrate of the β-galactosidase (reaction buffer). Finally, the stop solution was added, and the fluorescence was measured at 465 nm. All experiments were performed in triplicate, and are presented as the mean of four experiments.

### 4.6. eAC Redifferentiation

At the third passage, eAC were seeded into type I/III collagen sponges (Symatèse Biomatériaux, Chaponost, France) at a density of 8 × 10^5^ cells/sponges in the presence of 3D medium (DMEM HG, 2% FCS + ascorbic acid (A2p) (5 μL/mL) (Sigma-Aldrich, Saint-Louis, MO, USA) and antibiotics). These collagen sponges (100% of collagen, 2 mm thickness, 5 mm diameter, corresponding to a volume of 0.04 cm^3^, approximately 100 µm pore size) are composed of native type I collagen (90–95%) and type III collagen (5–10%) from calf skin; they are crosslinked with glutaraldehyde to increase their stability and then sterilized with β-radiation, and they do not swell after rehydration [30,89,90]. After 16 h of seeding, treatments with Promerim^®^ (30, 40, 60) were added at several concentrations (0.1, 0.5, 50, or 100 μg/mL) in the presence or absence of recombinant human BMP-2 (50 ng/mL, Inductos^®^) and/or Interleukin-1β (IL-1β; 10 ng/mL; Miltenyi Biotec Bergisch Gladbach, Germany). The media were renewed after three days of treatment. After seven days of incubation, the sponges were rinsed with PBS and placed at −80 °C until their protein or total RNA extractions.

### 4.7. RNA Isolation and RT-qPCR

Total RNAs were extracted using the TRIzol Reagent^®^ (Thermo Fisher Scientific, Waltham, MA, USA). One microgram of total RNA was reverse-transcribed into cDNA using iScript^®^ reverse transcript supermix (Bio-Rad, Hercules, CA, USA). Samples were diluted (1/100) in DEPC-water, and real-time PCR was performed on the CFX96 Touch (Bio-Rad, Hercules, CA, USA) using the “SYBR^®^ Green supermix” (Bio-Rad, Hercules, CA, USA). The sequences of the primers used are indicated in Supplementary Table S1. Relative gene expression was calculated using the 2^−ΔΔCt^ method expressed as the mean of triplicate samples. Each sample was normalized to *β-Actin* and *PPIA* house-keeping genes.

### 4.8. Western Blots

Sponges were crushed and lysed with RIPA containing a cocktail of protease inhibitors, as previously described [31]. The protein concentration was evaluated by a Bradford assay (Bio-Rad, Hercules, CA, USA). Then, 12 µg of total proteins were separated in 10% polyacrylamide gels containing 0.1% SDS, and transferred to a polyvinylidene membrane (PVDF, Bio-Rad, Hercules, CA, USA). Unspecific binding sites of the membranes were blocked with 10% non-fat milk powder in tris-buffered saline with 0.1% Tween (TBST) for 1 h. Then, membranes were incubated overnight at 4 °C with rabbit anti-human type I collagen (Novotec, Bron, France), rabbit anti-human type II collagen (Novotec, Bron, France), rabbit anti-human type IIB collagen (Covalab, Villeurbane, France), rabbit anti-human type X collagen (Abcam, Cambridge, UK), rabbit anti-human Htra1 (Merck Millipore, Billerica, MA, USA), rabbit anti-human GAPDH (Santa Cruz Biotechnology, Dallas, TX, USA), and mouse anti-human tubulin (Santa Cruz Biotechnology, Dallas, TX, USA). The following day, membranes were washed in TBST, and incubated with the secondary antibody (HRP-conjugated goat anti-rabbit or mouse IgG antibody (Jackson Immunoresearch; West Grove, PA, USA)). After a washing step, membranes were incubated with the Clarity Western ECL (Bio-Rad, Hercules, CA, USA) and signals visualized with the Chemidoc (Bio-Rad, Hercules, CA, USA).

### 4.9. Scratch Wound Assay

eAC were seeded in monolayers (P2) at a density of 20,000 cells per well in 96-well ImageLock plates (Essen BioScience, Michigan, USA) and were cultured for three days to form a confluent monolayer. A WoundMakerTM (Essen BioScience) was used to create uniform and reproducible wounds in all wells (four wells per condition) according to the manufacturer’s instructions. Then, the treatments were added, and the wound areas were monitored until 96 h, thanks to the IncuCyte ZOOM living cell imaging system (Essen BioScience). Wound surface area and cell confluence were measured using ImageJ software.

### 4.10. Statistical Analysis

All experiments were repeated at least four times with eAC from different horses. Values are presented as mean ± SD or as boxplots. Statistical analyses were performed using the Mann–Whitney U-test to determine significant differences between two groups, or using a two-way ANOVA followed by a Bonferonni test for multiple comparisons. Statistical analyses were done using Prism (Graphpad, San Diego, CA, USA). A *p*-value of ≤0.05 was considered to be significant.

## Data Availability

The data presented in this study are available in Supplementary Materials.

## Supplementary Materials

The following are available online at https://www.mdpi.com/1422-0067/22/2/580/s1, Figure S1: Promerim^®^ 30, 40, and 60 have no cytotoxic effect on equine articular chondrocytes in normoxia; Figure S2: Effect of low concentrations of Promerim^®^ 30, 40, and 60 on the mitochondrial activity of equine articular chondrocytes cultured in normoxia in the absence of serum; Figure S3: Effect of high concentrations of Promerim^®^ 30, 40, and 60 on the mitochondrial activity of equine articular chondrocytes cultured in normoxia in the absence of fetal calf serum; Figure S4: Effect of low concentrations of Promerim^®^ 30, 40, and 60 on the mitochondrial activity of equine articular chondrocytes cultured in normoxia in the presence of 2% fetal calf serum (FCS); Figure S5: Effect of high concentrations of Promerim^®^ 30, 40, and 60 on the mitochondrial activity of equine articular chondrocytes cultured in normoxia in the presence of 2% fetal calf serum (FCS); Figure S6: mRNA expression in equine articular chondrocytes treated with high concentrations Promerim^®^ 30 and 40 in the presence of BMP-2; Figure S7: mRNA expression in equine articular chondrocytes treated with Promerim^®^ 30 and 40 at high concentrations in the presence of both IL-1 and BMP-2; Figure S8: Complete gel and PVDF membranes analyzed in the western-blots; Figure S9: Under- and over-exposure of the blots presented in Figure 13; Figure S10: Complete gel and PVDF membranes analyzed in the western-blots; Figure S11: Under- and over-exposure of the blots presented in Figure 14; Table S1: Sequences of the primers used in RT-qPCR.
