# Supplementary material for: Marine Collagen Hydrolysates Downregulate the Synthesis of Pro-Catabolic and Pro-Inflammatory Markers of Osteoarthritis and Favor Collagen Production and Metabolic Activity in Equine Articular Chondrocyte Organoids
Source: Int J Mol Sci. 2021 Jan 8;22(2):580. doi: 10.3390/ijms22020580 (PMC7826754; doi:10.3390/ijms22020580)
Supplement: Supplementary file 1 [file ijms-22-00580-s001.pdf]

Marine collagen hydrolysates downregulate the synthesis of pro-catabolic and pro-inflammatory markers of osteoarthritis and favor collagen production and metabolic activity in equine articular chondrocyte organoids

Bastien Bourdon[1,2†], Romain Contentin[1†], Frédéric Cassé[1], Chloé Maspimby[1], Sarah Oddoux[2], Antoine Noël[2], Florence Legendre[1], Nicolas Gruchy[1,3] & Philippe Galéra[1*].

1. Normandie Univ, UNICAEN, BIOTARGEN, 14000 Caen, France.
2. Dielen laboratory, 50110 Tourlaville, France.
3. Department of Genetics, Normandy Center for Genomic and Personalized Medicine, Caen University Hospital, 14000 Caen, France.

[†]Contributed equally
*****Correspondence: philippe.galera@unicaen.fr

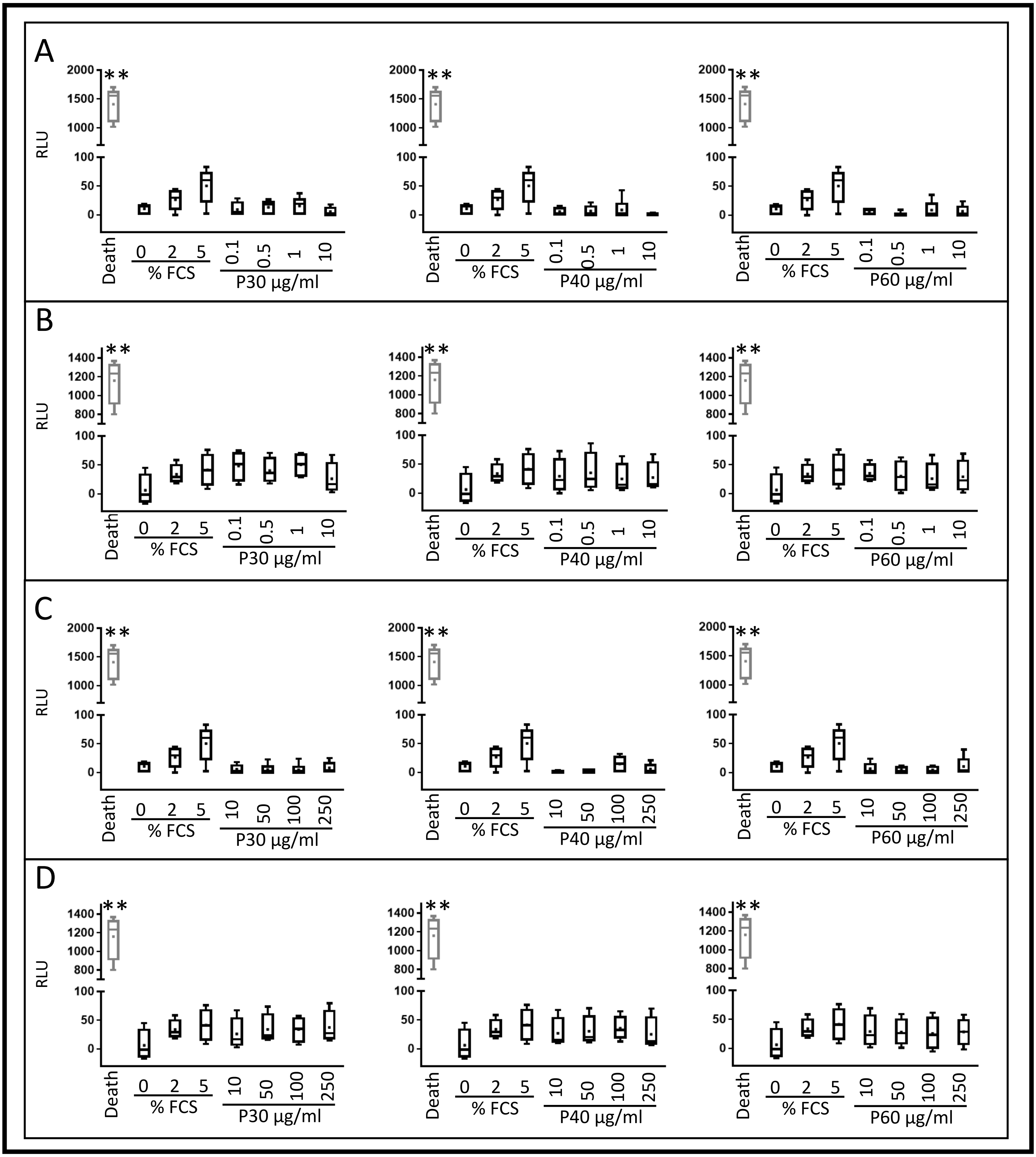

Figure S1: Promerim® 30, 40, and 60 have no cytotoxic effect on equine articular chondrocytes in normoxia. Equine articular chondrocytes were amplified and seeded at P3. At 80% of confluency, the cells were treated with Promerim® hydrolysates at several concentrations (0.1, 0.5, 1 and 10 µg/ml) (A and B) and (10, 50, 100 and 250 µg/ml) (C and D) in the absence of FCS (A and C) or the presence of 2% fetal calf serum (FCS) (B and D) and then cultured for 72 h in normoxia. Controls with 0, 2 and 5% of FCS were included, as well as a death control (triton-induced death). The levels of adenylate kinase were measured in the media after 72 h of culture (Toxilight kit, Interchim). Data are represented as box-plots (n= 5). Statistical analyses were performed using the Mann-Whitney test (* $p<0.05$) and the 0% and 2% FCS conditions were used as references. P30, P40 and P60: Promerim® 30, Promerim® 40 and Promerim® 60.

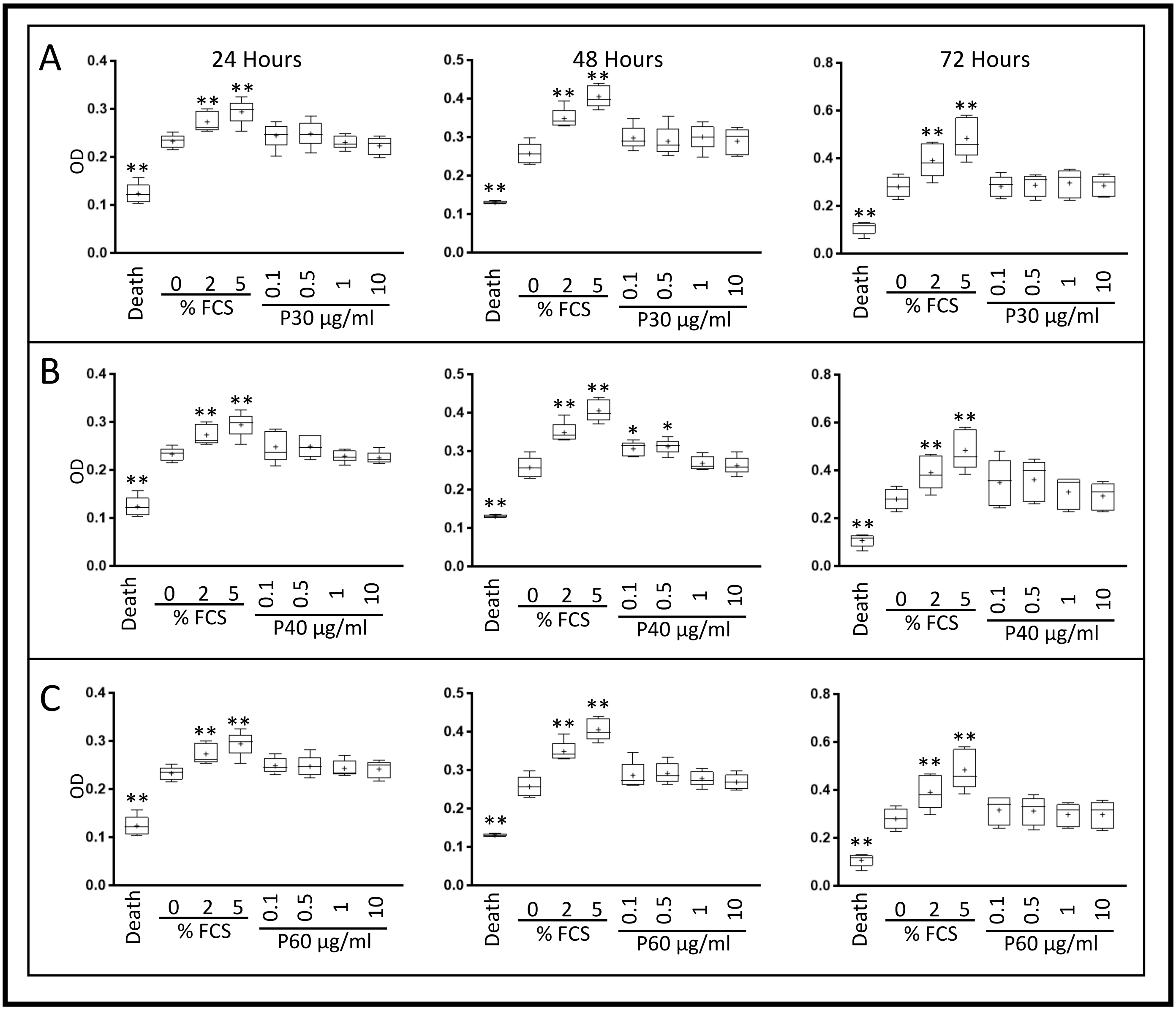

Figure S2: Effect of low concentrations of Promerim® 30, 40, and 60 on the mitochondrial activity of equine articular chondrocytes cultured in normoxia in the absence of serum.

Equine articular chondrocytes were amplified and seeded at P3. At 80% of confluency, the cells were treated with the Promerim® 30 (A), 40 (B), 60 (C) at several concentrations (0.1, 0.5, 1 and 10 µg/ml) in the absence of FCS and then cultured for 24, 48 and 72 h in normoxia. Controls with 0%, 2% and 5% of FCS, and a death control (triton-induced death) were included. The levels of formazan were measured (OD) in the media after 24, 48 and 72 h of culture (XTT kit, Roche). Data are represented as box-plots (n= 5). Statistical analyses were performed using the Mann-Whitney test (* $p<0.05$; ** $p<0.01$) and the 0% FCS condition was used as a reference. P30, P40 and P60: Promerim® 30, Promerim® 40 and Promerim® 60.

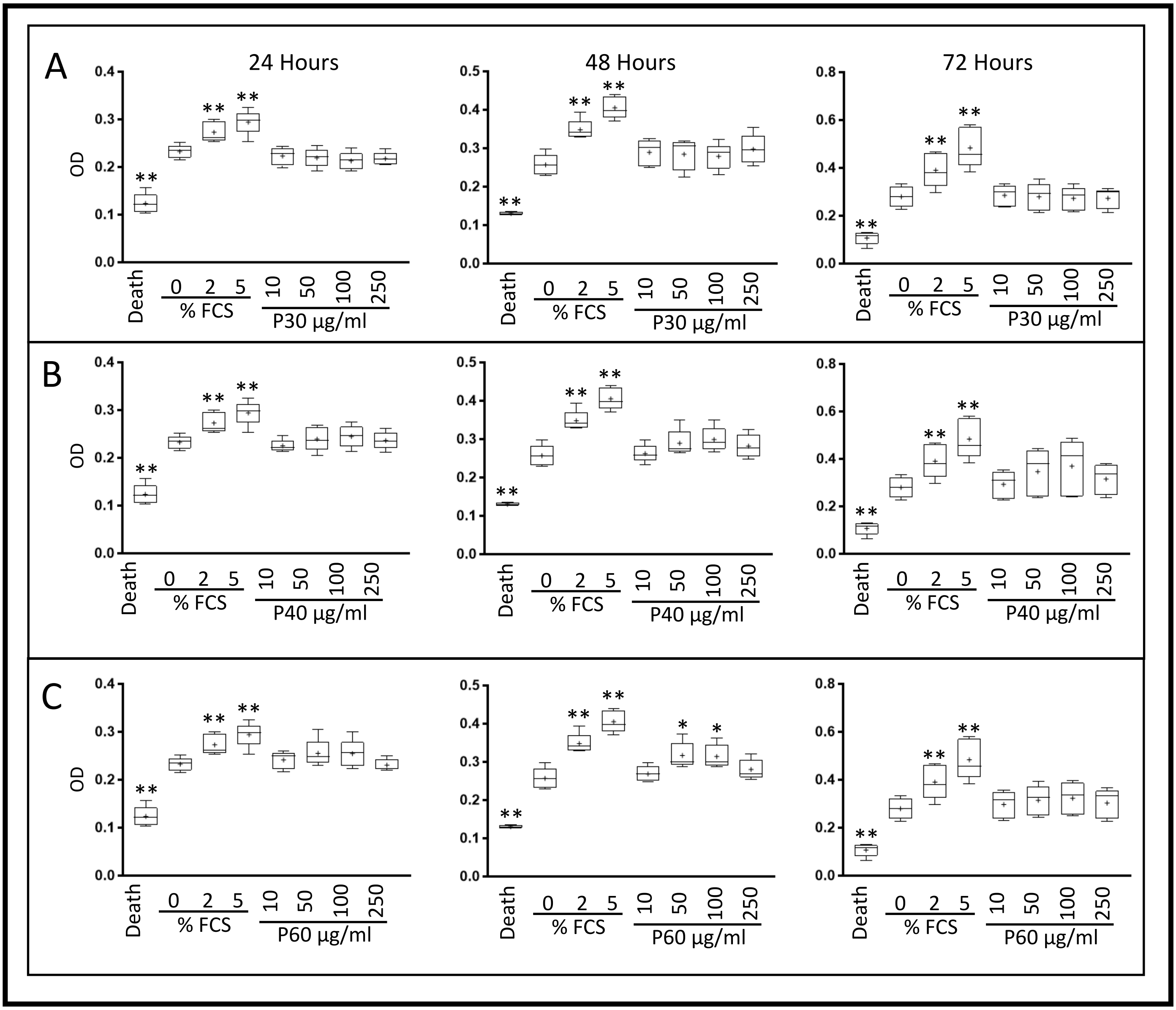

Figure S3: Effect of high concentrations of Promerim® 30, 40, and 60 on the mitochondrial activity of equine articular chondrocytes cultured in normoxia in the absence of fetal calf serum.

Equine articular chondrocytes were amplified and seeded at P3. At 80% of confluency, the cells were treated with Promerim® 30(A), 40 (B) and 60 (C) at several concentrations (10, 50, 100, 250 µg/ml) in the absence of FCS and then cultured for 24, 48 and 72 h in normoxia. Controls with 0%, 2% and 5% of FCS, and a death control (triton-induced death) were included. The levels of formazan were measured (OD) in the media at the end of the incubation period (XTT kit, Roche). Data are represented as box-plots (n= 5). Statistical analyses were based on the Mann-Whitney test (* p<0.05; ** p<0.01) and the 0% FCS condition was used as a reference. P30, P40 and P60: Promerim® 30, Promerim® 40 and Promerim® 60.

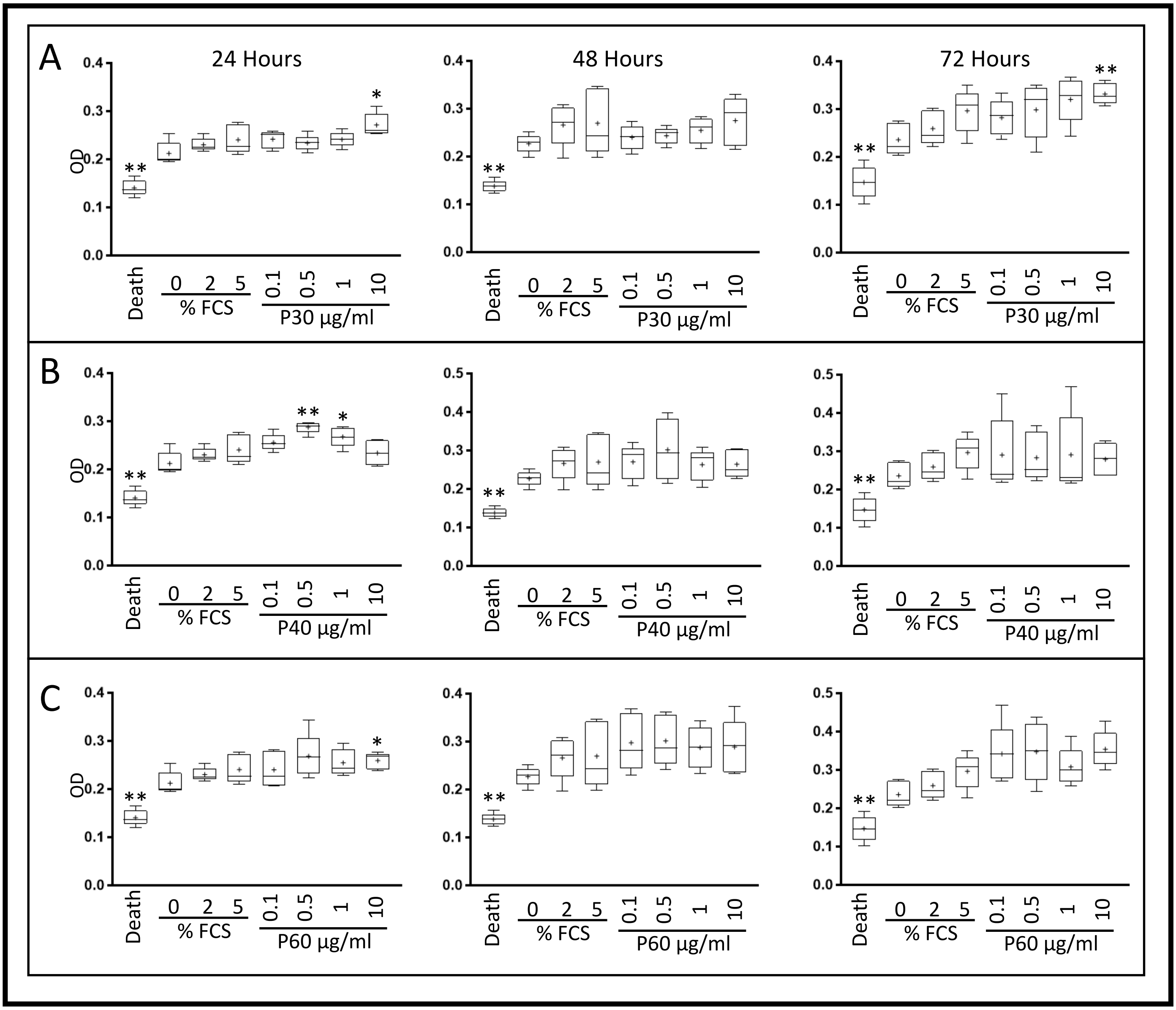

Figure S4: Effect of low concentrations of Promerim® 30, 40, and 60 on the mitochondrial activity of equine articular chondrocytes cultured in normoxia in the presence of 2% fetal calf serum (FCS).

Equine articular chondrocytes were amplified and seeded at P3. At 80% of confluency, the cells were treated with Promerim® 30 (A), 40 (B), 60 (C) at several concentrations (0.1, 0.5, 1 and 10 µg/ml) in the presence of 2% FCS and then cultured for 24, 48 and 72 h in normoxia. Controls with 0%, 2% and 5% of FCS, and a death control (triton-induced death) were included. The levels of formazan were measured (OD) in the media at the end of the culture period (XTT kit, Roche). Data are represented as box-plots (n= 5). Statistical analyses were based on the Mann-Whitney test (* $p<0.05$; ** $p<0.01$) and the 0% FCS condition was used as a reference. P30, P40 and P60: Promerim® 30, Promerim® 40 and Promerim® 60.

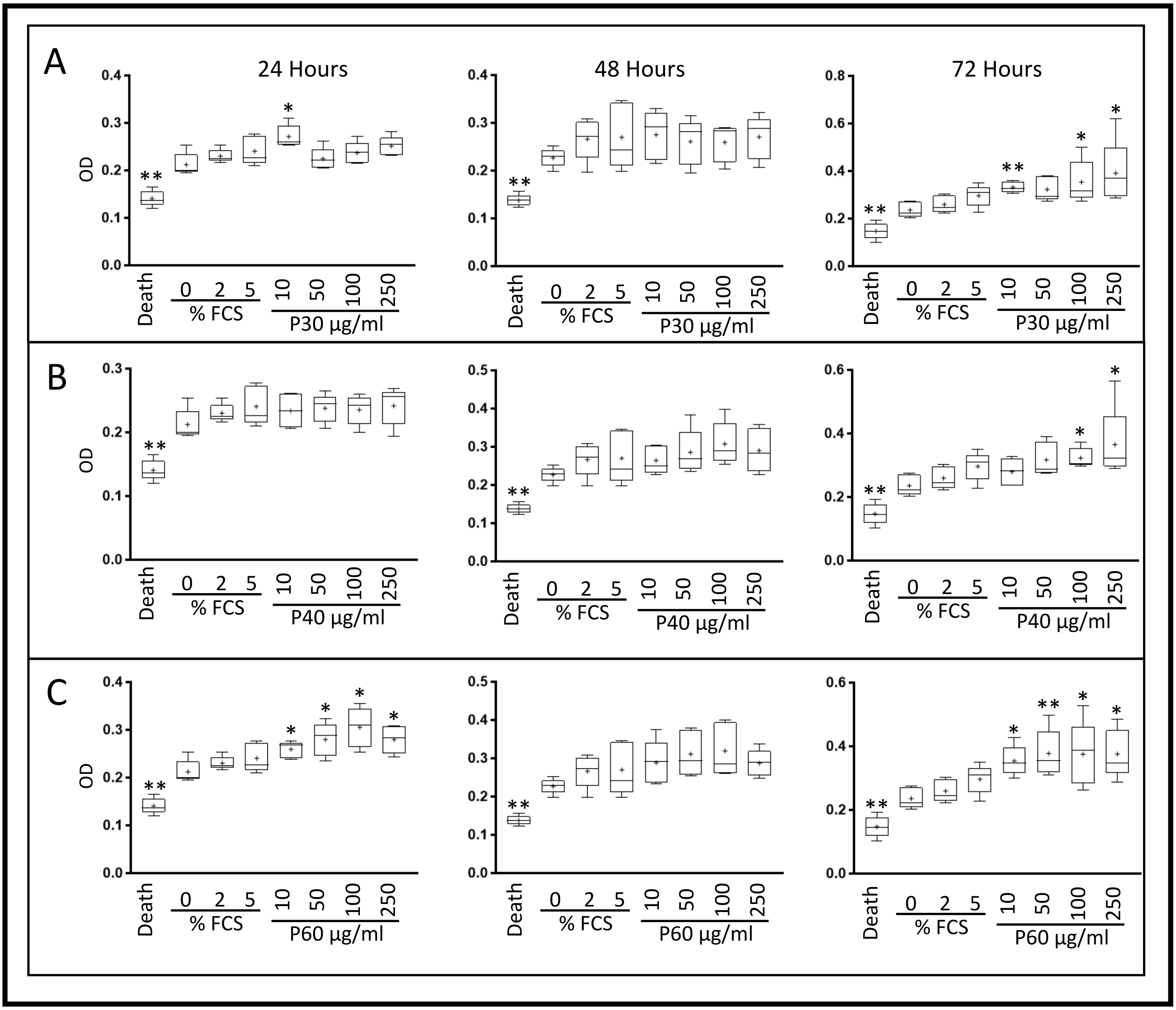

Figure S5: Effect of high concentrations of Promerim® 30, 40, and 60 on the mitochondrial activity of equine articular chondrocytes cultured in normoxia in the presence of 2% fetal calf serum (FCS).

Equine articular chondrocytes were amplified and seeded at P3. At 80% of confluency, the cells were treated with the Promerim® 30 (A), 40 (B), 60 (C) at several concentrations (10, 50, 100, 250 µg/ml) in the presence of 2% of FCS and then cultured for 24, 48 and 72 h in normoxia. Controls with 0%, 2% and 5% of FCS, and a death control (triton-induced death) were included. The levels of formazan were measured (OD) in the media at the end of the incubation period (XTT kit, Roche). Data are represented as box-plots (n= 5). Statistical analyses were based on the Mann-Whitney test (* p<0.05; ** p<0.01) and the condition 2% FCS was used as a reference. P30, P40 and P60: Promerim® 30, Promerim® 40 and Promerim® 60.

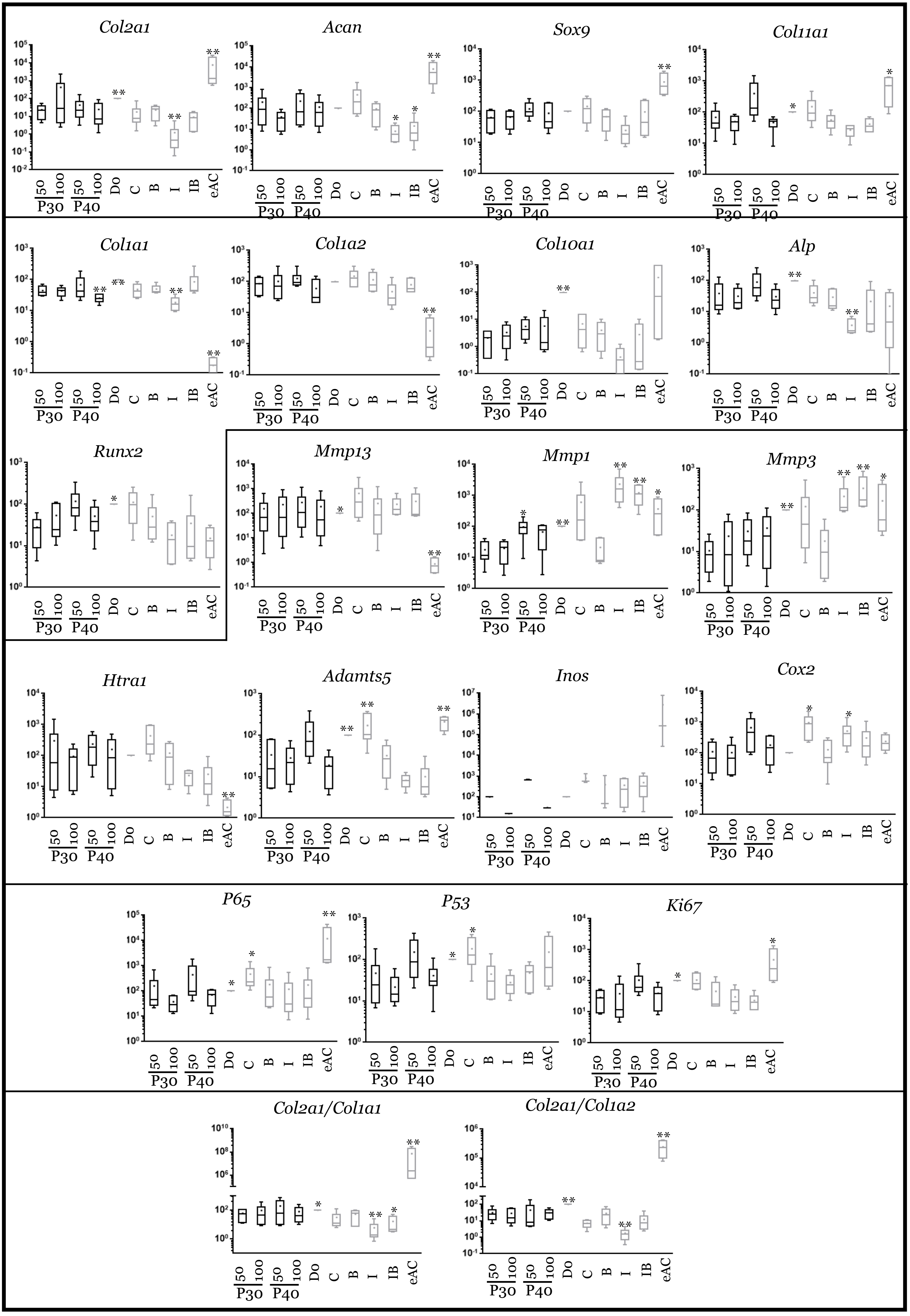

Col2a1
Acan
Sox9
Col11a1
Col1a1
Col1a2
Col10a1
Alp
Runx2
Mmp13
Mmp1
Mmp3
Htra1
Adamts5
Inos
Cox2
P65
P53
Ki67
Col2a1/Col1a1
Col2a1/Col1a2
50
100
50
100
Do
C
B
I
IB
eAC
P30
P40

Figure S6: mRNA expression in equine articular chondrocytes treated with high concentrations Promerim® 30 and 40 in the presence of BMP-2.

Equine articular chondrocytes were grown in type I/III collagen sponges at the third passage (P3). They were incubated for 7 days under hypoxia in the absence (C: control) or both the presence of BMP-2+P30 and BMP-2+P40, or BMP-2 alone (B), or IL-1 alone (I), or BMP-2 and IL-1 (IB). The Promerim® were used at the concentrations of 50 and 100 µg/ml. The mRNAs were estimated using RT-qPCR after normalization with respect to the β-actin reference gene. Transcripts expression is shown in arbitrary units. The *Col2a1:Col1a1* and *Col2a1:Col1a2* ratios are given. The results are shown as box-plots (median, quartiles, extreme values) and the significance of the values between the different treatments and the control case (BMP-2) was tested using a Mann-Whitney test (* $p < 0.05$; ** $p < 0.01$); n= 6. eAC: mRNA extracts obtained from equine articular chondrocytes released from cartilage after overnight enzymatic digestion were used as controls. D0: cells seeded in sponges and arrested after 16 h of incubation. P30 and P40: Promerim® 30 and Promerim® 40.

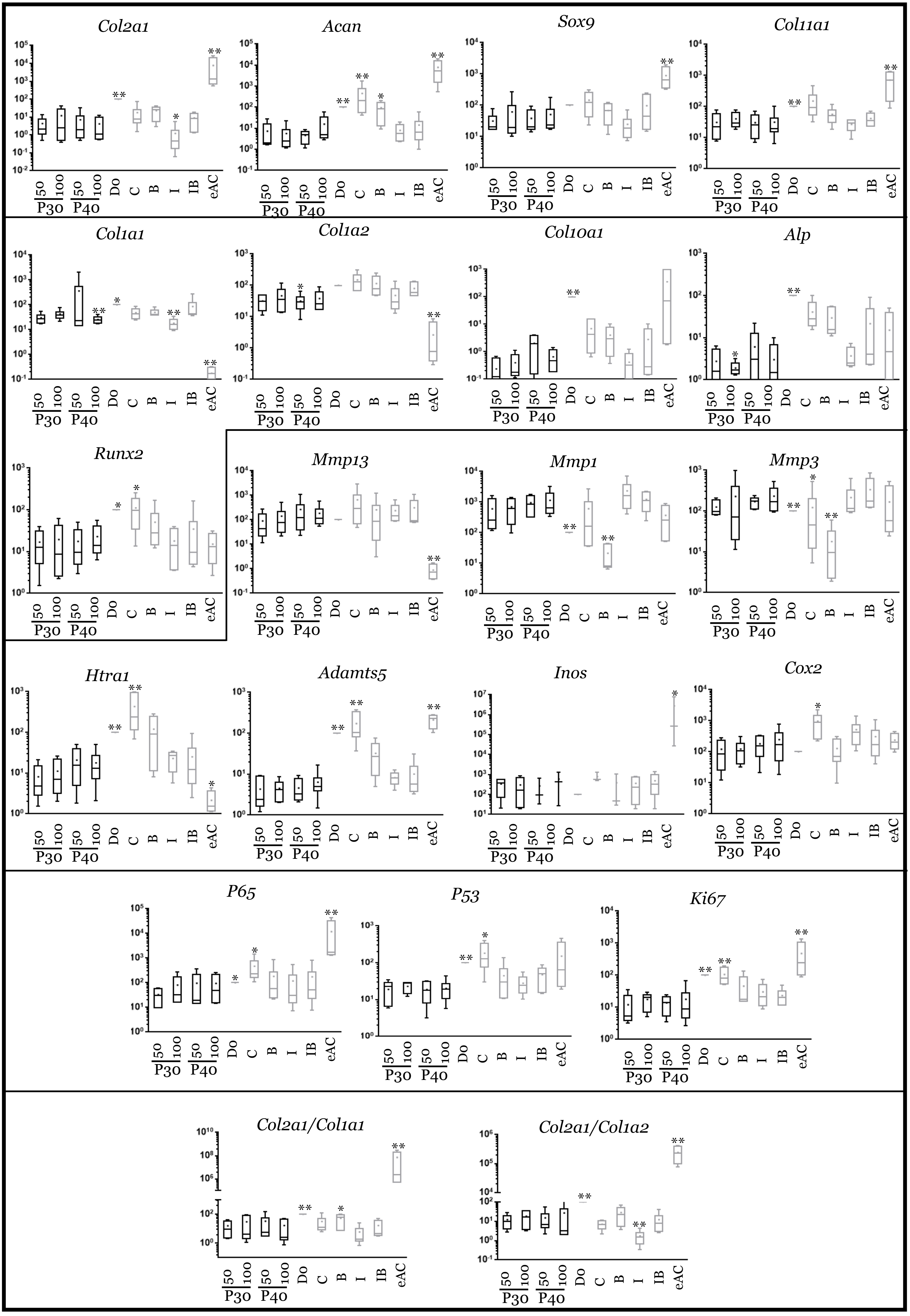
Col2a1
Acan
Sox9
Col11a1
Col1a1
Col1a2
Col10a1
Alp
Runx2
Mmp13
Mmp1
Mmp3
Htra1
Adamts5
Inos
Cox2
P65
P53
Ki67
Col2a1/Col1a1
Col2a1/Col1a2
50
100
50
100
P30
P40
Do
C
B
I
IB
eAC

Figure S7: mRNA expression in equine articular chondrocytes treated with Promerim® 30 and 40 at high concentrations in the presence of both IL-1 and BMP-2.

Equine articular chondrocytes were grown in type I/III collagen sponges at the third passage (P3). They were incubated during 7 days under hypoxia in the absence (C: control) or both the presence of P30+IL-1+BMP-2 and P40+IL-1+BMP-2, or BMP-2 alone (B), or IL-1 alone (I), or BMP-2 together with IL-1 (IB). The Promerim® were used at the concentrations of 50 and 100 µg/ml. The mRNAs were estimated using RT-qPCR after normalization with respect to the β-actin reference gene. Transcript expression is shown in arbitrary units. The *Col2a1*:*Col1a1* and *Col2a1*:*Col1a2* ratios are given. The results are shown as box-plots (median, quartiles, extreme values) and the significance of the values between the different treatments and the control case (IL-1+BMP-2) was tested using a Mann-Whitney test (*$p < 0.05$; **$p < 0.01$); n= 3. eAC: mRNA extracts obtained from equine articular chondrocytes released from cartilage after overnight enzymatic digestion were used as controls. D0: cells seeded in sponges and arrested after 16 h of incubation. P30 and P40: Promerim®30 and 40.

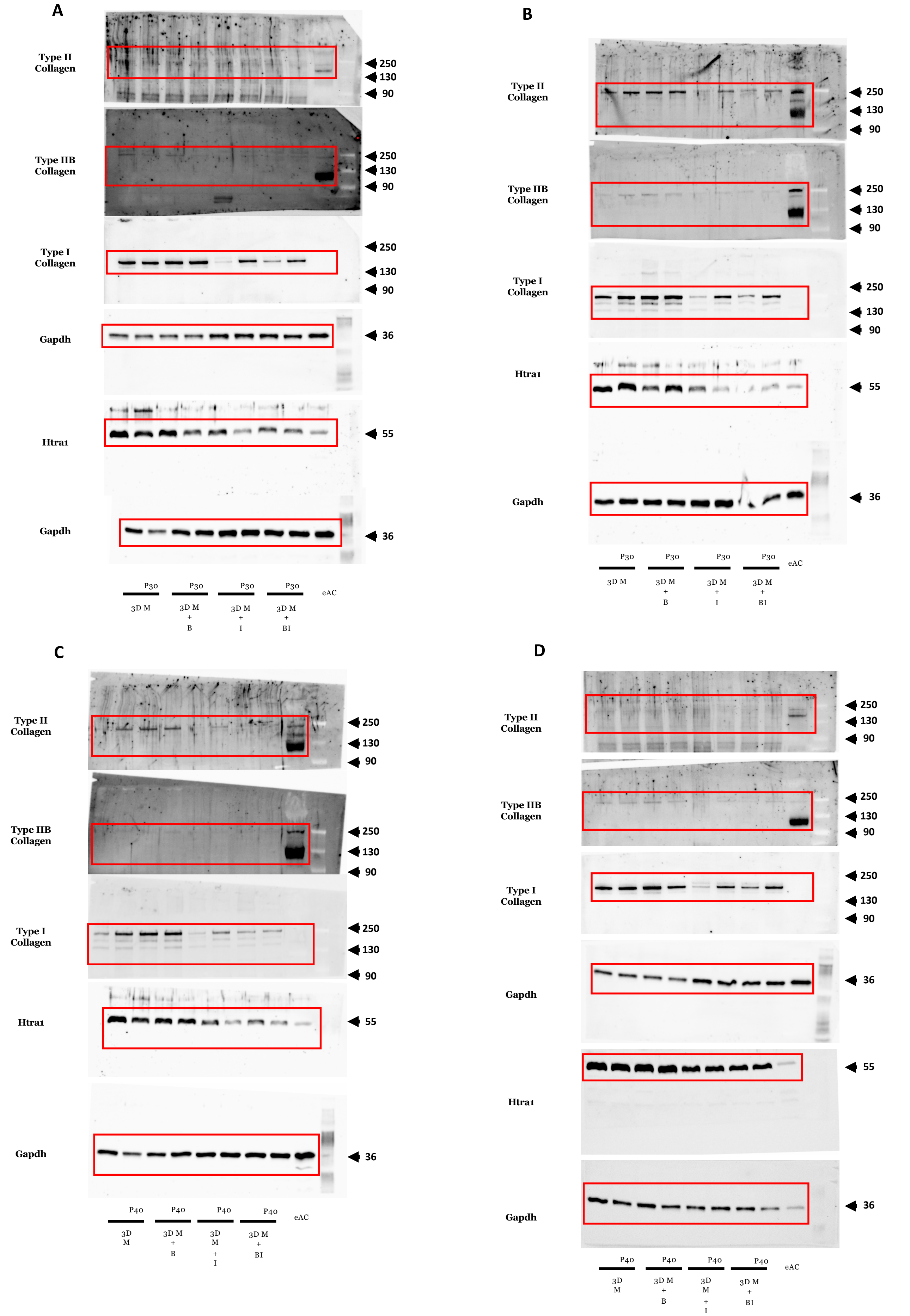

Figure S8: Complete gel and PVDF membranes analyzed in the western- blots.

For the western-blots presented in figure 13, respectively A, B, C and D, the complete images of the membranes captured with Chemidoc MP Imaging System (Bio-Rad) are shown. The molecular weight marker (MW kDa) is indicated on the right for the images shown. The cropped images are highlighted in the red lines.

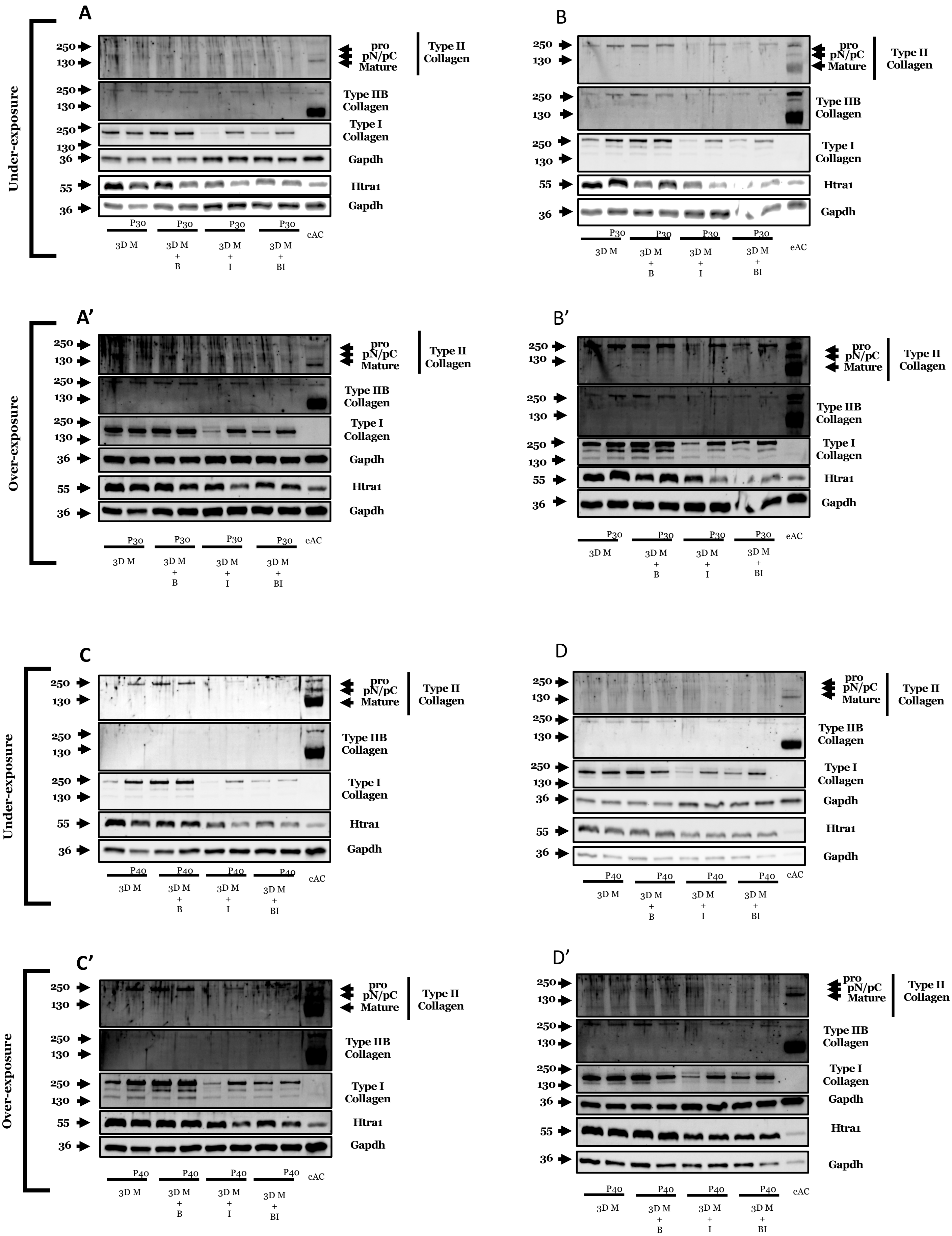

Figure S9: Under- and over-exposure of the blots presented in figure 13.

Under-exposure (A, B, C, D) and over-exposure (A’, B’, C’, D’) are shown. The molecular weight marker (MW kDa) is indicated on the left for the images presented.

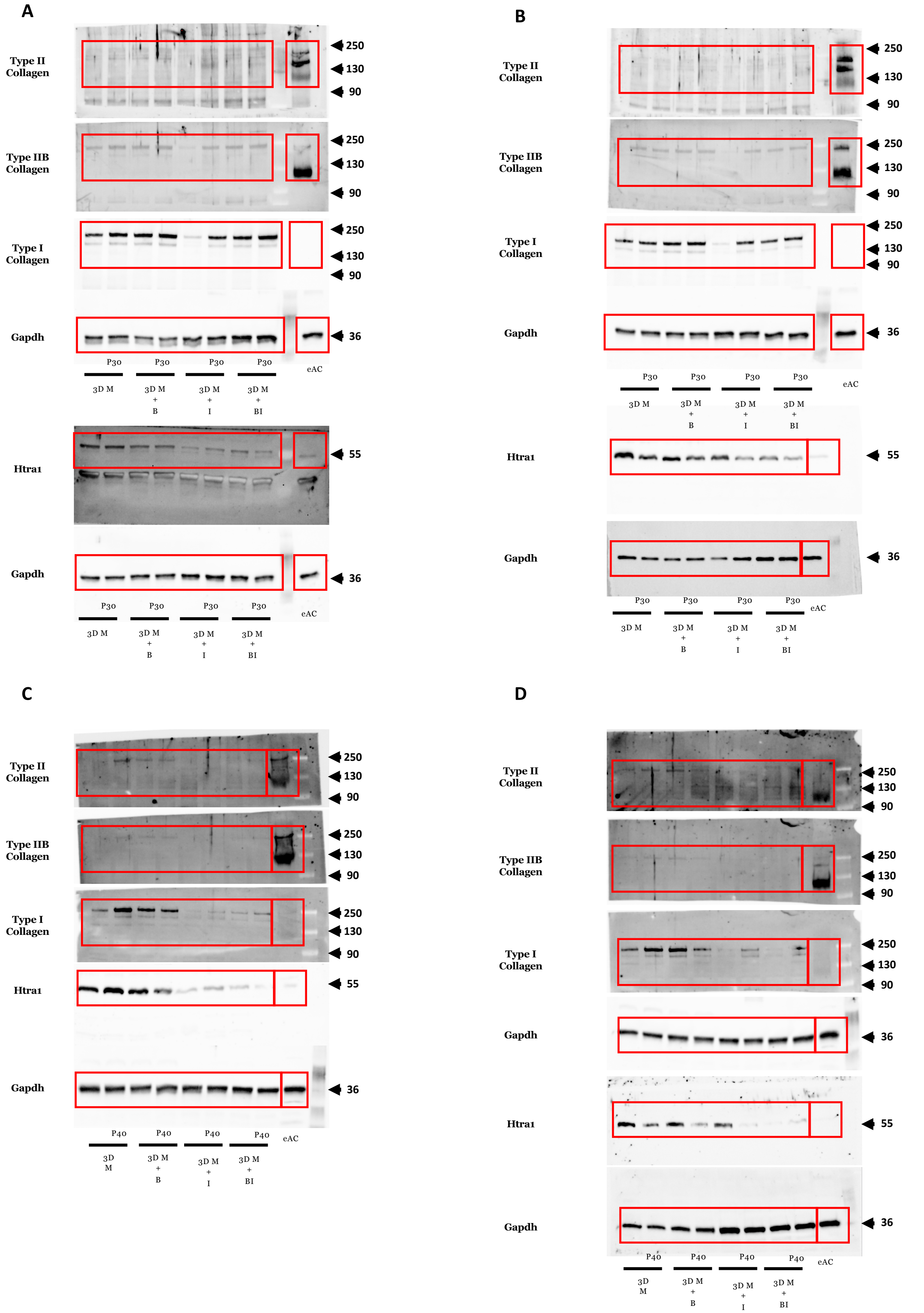

Figure S10: Complete gel and PVDF membranes analyzed in the western- blots.
For the western-blots presented in figure 14, respectively A, B, C and D, the complete images of the membranes captured with Chemidoc MP Imaging System (Bio-Rad) are shown. The molecular weight marker (MW kDa) is indicated on the right for the images shown. The cropped images are highlighted in the red lines.

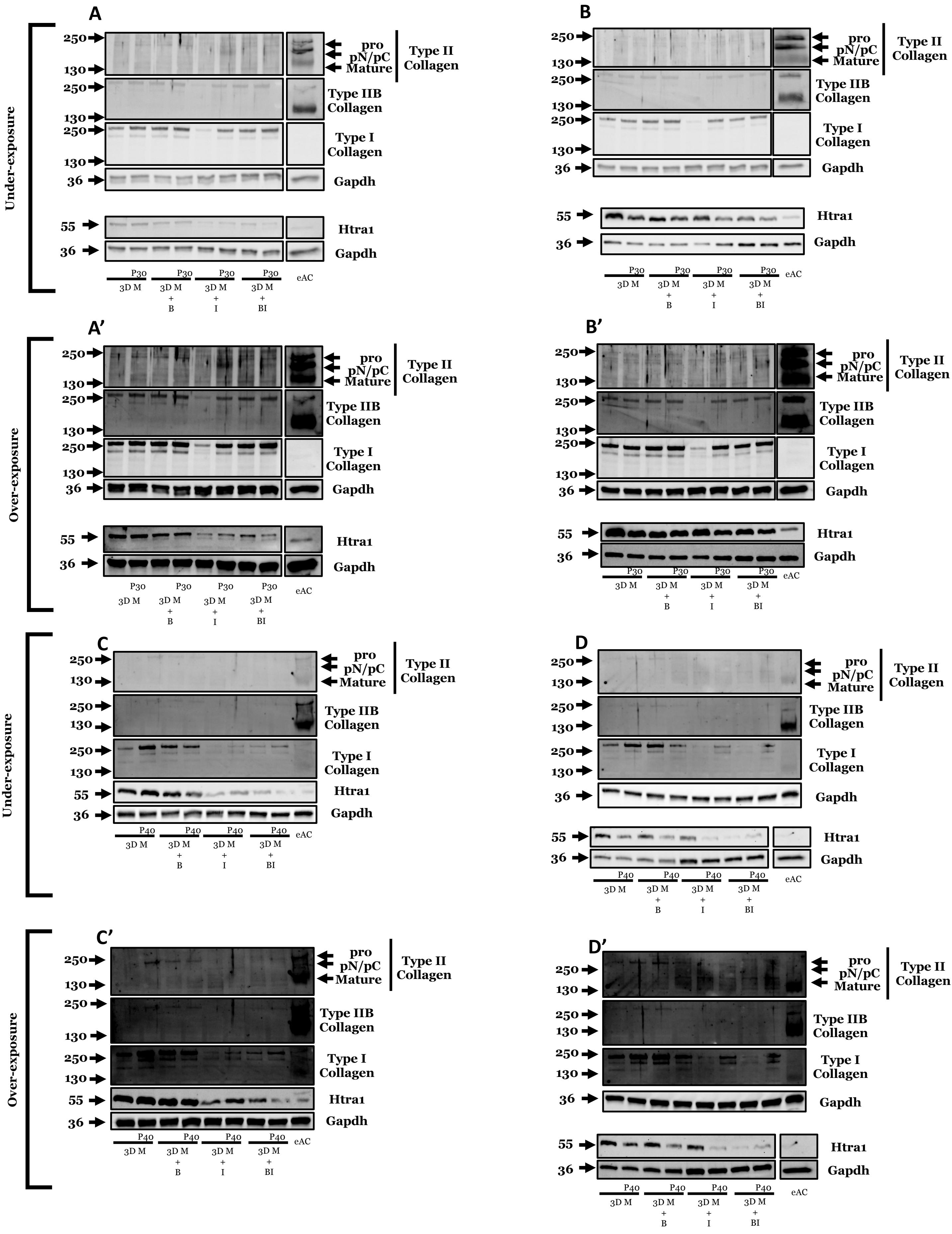

Figure S11: Under- and over-exposure of the blots presented in figure 14.

Under-exposure (A, B, C, D) and over-exposure (A′, B′, C′, D′) are shown below. The molecular weight marker (MW kDa) is indicated on the left for the images presented.

Gene	Forward sequence	Reverse sequence
Acan	TGT CAA CAA CAA TGC CCA AGA C	CTT CTT CCG CCC AAA GGT CC
Β-Actin	GAT GAT GAT ATC GCC GCG CTC	TGC CCC ACG TAT GAG TCC TT
Adamts5	AAG GGA CAC CAT GTG GCA AA	CCC ACA TGA GCG AGA ACA CT
Alpl	GAC ATG ACC TCC CAG GAA GA	GCA GTG AAG GGC TTC TTG TC
Col1a1	TGC CGT GAC CTC AAG ATG TG	CGT CTC CAT GTT GCA GAA GA
Col1a2	CCA GAG TGG AGC AGC GGT TA	GGG ATG TTT TCA GGT TGA GCC
Col2a1	GGC AAT AGC AGG TTC ACG TAC A	CGA TAA CAG TCT TGC CCC ACT T
Col10a1	GCA CCC CAG TAA TGT ACA CCT ATG	GAG CCA CAC CTG GTC ATT TTC
Col11a1	TTG CTG ATG GGA AGT GGC AT	GCT GCT TTG GGG TCA CCT AT
Cox2	CGA GGT CCA GCT TTC ACC A	GCG GAT ACA CCT CGC CAT T
Htra1	GGA CTT CAT GTT TCC CTC AA	GTT CTG CTG AAC AAG CAA CA
Inos	TTT GGC TGG TCC CCC GAT TT	GCC AGC GTT TCC GAT TTT CC
Ki67	AAG CTG CAC GTT CAT GGA GA	ACC CAC AGT TCT TCC TCC GA
Mmp1	CGA AGG GAA CCC TCG GTG GGA	TGG CCT GGT CCA CAT CTG CTC
Mmp3	GAG GAA ATG AGG AAC AAG CGG	GAG GGA AAC CCA GAG TGT GGA
Mmp13	TGA AGA CCC GAA CCC TAA ACA T	GAA GAC TGG TGA TGG CAT CAA G
P53	CAC CTG AGG TTG GCT CTG AC	GCA CAA ACA CGC ACC TCA AA
P65	CAC GGA TAC CAC CAA GAC CC	GTC TGG ATG CGC TGA CTG AT
Ppia	CCC TAC CGT GTT CTT CGA CA	GTG AAG TCA CCA CCC TGA CA
Runx2	GCA GTT CCC AAG CAT TTC AT	CAC TCT GGC TTT GGG AAG AG
Sox9	CAA GAA GGA CCA CCC GGA CTA	GGA GAT GTG TGT CTG CTC CGT

Supplementary table 1. Sequences of the primers used in RT-qPCR.
